# From offshore to onshore probabilistic tsunami hazard assessment via efficient Monte Carlo sampling

**DOI:** 10.1093/gji/ggac140

**Published:** 2022-04-11

**Authors:** Gareth Davies, Rikki Weber, Kaya Wilson, Phil Cummins

**Affiliations:** Place, Space and Communities Division, Geoscience Australia, Canberra ACT 2601, Australia; Place, Space and Communities Division, Geoscience Australia, Canberra ACT 2601, Australia; Research School of Earth Sciences, Australian National University, Acton, ACT 0200, Australia; Place, Space and Communities Division, Geoscience Australia, Canberra ACT 2601, Australia; Place, Space and Communities Division, Geoscience Australia, Canberra ACT 2601, Australia; Research School of Earth Sciences, Australian National University, Acton, ACT 0200, Australia

**Keywords:** Tsunamis, Numerical approximations and analysis, Statistical methods

## Abstract

Offshore Probabilistic Tsunami Hazard Assessments (offshore PTHAs) provide large-scale analyses of earthquake-tsunami frequencies and uncertainties in the deep ocean, but do not provide high-resolution onshore tsunami hazard information as required for many risk-management applications. To understand the implications of an offshore PTHA for the onshore hazard at any site, in principle the tsunami inundation should be simulated locally for every earthquake scenario in the offshore PTHA. In practice this is rarely feasible due to the computational expense of inundation models, and the large number of scenarios in offshore PTHAs. Monte Carlo methods offer a practical and rigorous alternative for approximating the onshore hazard, using a random subset of scenarios. The resulting Monte Carlo errors can be quantified and controlled, enabling high-resolution onshore PTHAs to be implemented at a fraction of the computational cost. This study develops efficient Monte Carlo approaches for offshore-to-onshore PTHA. Modelled offshore PTHA wave heights are used to preferentially sample scenarios that have large offshore waves near an onshore site of interest. By appropriately weighting the scenarios, the Monte Carlo errors are reduced without introducing bias. The techniques are demonstrated in a high-resolution onshore PTHA for the island of Tongatapu in Tonga, using the 2018 Australian PTHA as the offshore PTHA, while considering only thrust earthquake sources on the Kermadec-Tonga trench. The efficiency improvements are equivalent to using 4–18 times more random scenarios, as compared with stratified-sampling by magnitude, which is commonly used for onshore PTHA. The greatest efficiency improvements are for rare, large tsunamis, and for calculations that represent epistemic uncertainties in the tsunami hazard. To facilitate the control of Monte Carlo errors in practical applications, this study also provides analytical techniques for estimating the errors both before and after inundation simulations are conducted. Before inundation simulation, this enables a proposed Monte Carlo sampling scheme to be checked, and potentially improved, at minimal computational cost. After inundation simulation, it enables the remaining Monte Carlo errors to be quantified at onshore sites, without additional inundation simulations. In combination these techniques enable offshore PTHAs to be rigorously transformed into onshore PTHAs, with quantification of epistemic uncertainties, while controlling Monte Carlo errors.

## 1 INTRODUCTION

Tsunami inundation is rare on most coastlines, but large events can have catastrophic impacts (Okal 2011; Chock *et al*. 2013; Okal 2015). Limited data is usually available to constrain the frequency of inundation, leading to uncertainties in the hazard that are consequential for risk analysis and management (Fukutani *et al*. 2018; Løvholt *et al*. 2019; Wood *et al*. 2020; Tonini *et al*. 2021). Probabilistic Tsunami Hazard Assessment (PTHA) offers a structured approach to quantifying the hazard and associated uncertainties, while integrating data, physical and statistical models and expert judgements (Grezio *et al*. 2017; Behrens *et al*. 2021). Although PTHA is not yet standardized, several large-scale PTHAs provide analyses of offshore earthquake-tsunami frequencies and uncertainties at national-to-global scales (e.g. Burbidge *et al*. 2008, 2009; Chock 2015; Power *et al*. 2017; Davies & Griffin 2018; Basili *et al*. 2021). Offshore PTHAs are well suited to representing diverse earthquake-tsunami sources at large spatial scales, and describing epistemic uncertainties (i.e. due to lack of knowledge) in tsunami frequencies and source processes. But offshore PTHAs cannot accurately represent tsunami shoaling and inundation, so provide limited information on the onshore hazard. It is challenging to transform offshore PTHAs into high-resolution onshore PTHAs that give a similarly comprehensive representation of the hazard and uncertainty, while resolving spatial scales on the order of 10 m that are relevant to risk-management (Lynett *et al*. 2016). This is primarily because tsunami inundation modelling is computationally expensive, and a wide-range of scenarios are significant for characterizing the hazard (Lorito *et al*. 2015; Sepúlveda *et al*. 2019; Gibbons *et al*. 2020; Tonini *et al*. 2021).

Recent offshore PTHAs represent hypothetical earthquake-tsunamis with a large set of scenarios *E*, containing on the order of 10^5^−10^7^ individual scenarios *e* ∈ *E* (Davies & Griffin 2020; Basili *et al*. 2021; Tonini *et al*. 2021). For each scenario the tsunami is simulated using computationally cheap models that are accurate in deep-water far from the coast, but inaccurate near the coast due to coarse resolution and neglect of non-linearity (e.g. Burbidge *et al*. 2008; Miranda *et al*. 2014; Lynett *et al*. 2016; Molinari *et al*. 2016; Davies 2019). Scenario-frequencies are represented using a family of models *I* which collectively represent epistemic uncertainties in tsunami occurrence-rates, with each model *i* ∈ *I* assigning a long-term average rate *r_i_*(*e*) (events/year) to every scenario (e.g. Power *et al*. 2012; Davies & Griffin 2020; Basili *et al*. 2021). For any quantity of interest *Q*, such as the tsunami maxima at a particular site, this leads to a family of exceedance-rate curves describing the hazard and uncertainties. If scenarios do not overlap in time, the long-term average rate at which *Q* exceeds a threshold *Q*^*T*^ under scenario-frequency model *i* is denoted *λ_i_*(*Q* > *Q*^*T*^) (events/year) and:

(1) $$\begin{equation*}
\lambda _{i}\left(Q \gt Q^{T} \right) = \sum _{e \in E} \mathbb {1} _{(Q(e)\gt Q^{T})} r_{i}(e) .
\end{equation*}
$$

Here $\mathbb {1}_{x}$ is the indicator function (=  1 if *x* is true and 0 otherwise). If the offshore PTHA describes an infinite set of scenarios then eq. (1) becomes an integral (Behrens *et al*. 2021). But herein we assume a large finite set of scenarios is used, and eq. (1) gives the offshore PTHA’s exceedance-rate curves.

At coastal sites, the offshore PTHA is not designed to simulate the tsunami, but exceedance-rate curves can in-principle be derived from eq. (1) by using a site-specific hydrodynamic model to compute *Q*(*e*) for all scenarios. However for most onshore hazard assessments this is too computationally expensive, especially at large spatial scales. Recent studies using state-of-the-art high-performance-computing simulate tens of thousands of inundation scenarios over tens of kilometres of coastline at approximately 10 m resolution (Gibbons *et al*. 2020; Tonini *et al*. 2021), and most studies are conducted with far fewer computational resources. Furthermore a long-term goal of tsunami hazard research is the quantification of inundation hazards at much larger spatial scales (national or even global), for which the computational demands will be greater. Thus it is important to have good techniques to compute eq. (1), perhaps approximately, at lesser computational expense. This is the focus of this study.

In hazard assessment practice, often a small number of scenarios are selected from the offshore PTHA, having tsunami maxima corresponding to a specified exceedance-rate (e.g. 1/500 or 1/2500) near the coastal site of interest (e.g. Boswood 2013; Cardno 2013; Chock 2016). The exceedance-rates are often inferred from the mean scenario-frequency model or the 84th percentile (Leonard *et al*. 2008; MCDEM 2016; Tonini *et al*. 2021). This is a pragmatic approach when only a small number of inundation scenarios can be simulated. But there is no guarantee that the resulting inundation will closely approximate the exact solution, derived from eq. (1) by simulating inundation for every scenario. This is because modelled offshore wave heights are not monotonically related to modelled nearshore wave heights or inundation; relationships between these variables exhibit substantial scatter in practice (e.g. Tang *et al*. 2009; Mueller *et al*. 2015). For this reason this study focusses on more rigorous approximations to eq. (1) at coastal sites, which enable the offshore PTHA to be translated onshore with controlled error, while having much lower computational cost than required to simulate inundation for all scenarios.

There are two complementary approaches to solving eq. (1) at coastal sites, and thus translating offshore PTHAs onshore:

- Develop efficient methods to compute *Q*(*e*) using high-performance-computing and/or approximation methods (e.g. de la Asunción *et al*. 2013; de Baar & Roberts 2017; Arce Acuña & Aoki 2018; Guillas *et al*. 2018; Williamson *et al*. 2020; Fukutani *et al*. 2021; Giles *et al*. 2021);
- Develop techniques to approximate eq. (1) that require inundation simulations for only a small fraction of all scenarios (e.g. Lorito *et al*. 2015; Sepúlveda *et al*. 2017, 2019; Volpe *et al*. 2019).

While both are of fundamental importance to PTHA, this study focusses only on the second approach. We consider Monte Carlo approximations of eq. (1) that only require simulating inundation for a (weighted) random subset of scenarios. Our approach builds upon stratified-sampling, in which scenarios are binned by magnitude and a fixed number are randomly sampled from each bin (e.g. De Risi & Goda 2017; Williamson *et al*. 2020; Basili *et al*. 2021; Zamora *et al*. 2021). This standard approach is improved by leveraging the offshore PTHA to automatically focus sampling on scenarios with larger waves near the site of interest, and on magnitude-bins that contribute most to Monte Carlo errors.

Previously Lorito *et al*. (2015) and Volpe *et al*. (2019) developed techniques that leverage offshore PTHA wave-heights to guide onshore PTHA scenario sampling. They firstly discarded scenarios that produced small waves offshore of the site of interest. The remaining scenarios were clustered by offshore wave-heights, with each cluster represented using one inundation simulation (Lorito *et al*. 2015). The clustering step was later refined to discard infrequent scenarios, and to reduce bias for near-field tsunamis (Volpe *et al*. 2019). Sepúlveda *et al*. (2017) also considered the problem of selecting a good subset of scenarios from among random earthquakes, although they did not assume the existence of offshore PTHA waveforms. Their Stochastic Reduced Order Method (SROM) enables selecting and weighting a subset of scenarios that collectively represents the variability of the coseismic displacement. A case-study suggested this leads to a more efficient representation of the tsunami variability, compared with standard Monte Carlo sampling (Sepúlveda *et al*. 2017).

Compared with previous works, our study places greater emphasis on simple analytical techniques to estimate errors caused by the limited sampling of scenarios, both before and after detailed inundation simulations are undertaken. This serves two purposes:

- Before inundation simulation, it facilitates testing and refinement of Monte Carlo sampling schemes. This enables poorly performing schemes to be identified and fixed, at minimal computational cost.
- After inundation simulation, Monte Carlo errors can be estimated at onshore sites, without additional simulations. Thus the numerical reliability of the onshore hazard can be easily quantified.

Used in combination, these techniques facilitate the control of Monte Carlo errors in onshore hazard assessments.

This paper is structured as follows. Section 2 introduces the site of interest and the offshore PTHA used to demonstrate our approach (Davies & Griffin 2018, 2020). Note our methodology is not specific to this offshore PTHA. Section 3 reviews common Monte Carlo integration techniques for offshore-to-onshore PTHA: synthetic catalogues (Section 3.1) and stratified-sampling by magnitude (Section 3.2). Methods are presented to cheaply estimate the accuracy in coastal hazard applications, and we assess the computational cost of using many scenario-frequency models *i* ∈ *I* to represent epistemic uncertainties. Section 3.3 extends the analysis to a novel Monte Carlo integration technique, termed ‘stratified/importance-sampling’ herein, which leverages information from the offshore PTHA to better sample scenarios featuring larger waves near the site of interest. Section 3.4 develops techniques to guide non-uniform sampling of magnitude-bins, also informed by the offshore PTHA, which lead to additional efficiency improvements for both stratified and stratified/importance-sampling. Section 3.5 tests a particular combination of these approaches at offshore sites in the context of our inundation hazard application, which is presented in Section 4. Section 4.1 presents the high-resolution tsunami model and comparison with historic tsunamis at our site of interest, while Section 4.2 tests that Monte Carlo results obtained from the high-resolution model are consistent with the offshore PTHA at a deep-ocean site. Section 4.3 examines onshore hazard results with a focus on the quantification of Monte Carlo errors and epistemic uncertainties.

## 2 STUDY SITE AND OFFSHORE PTHA

**Figure 1.**
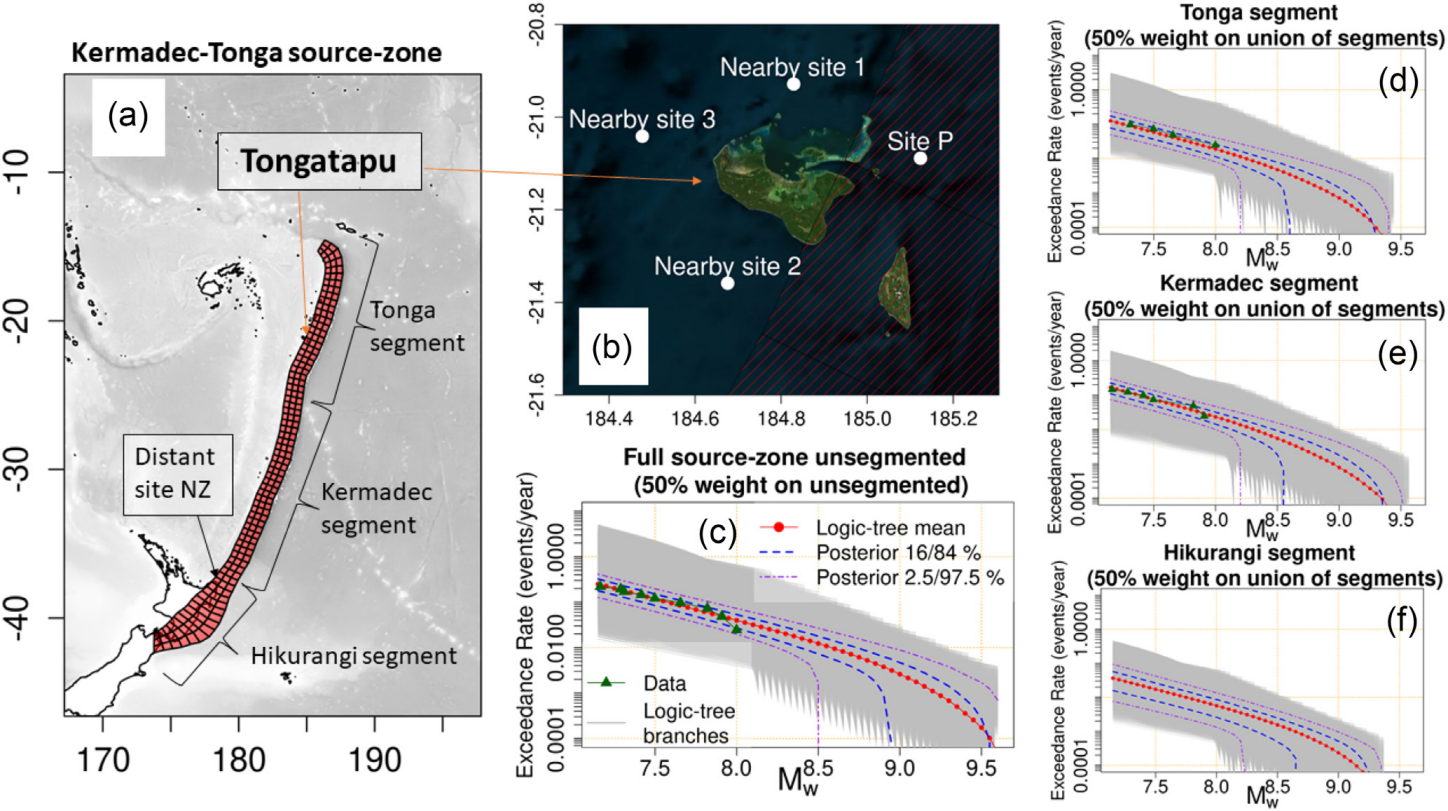
Tongatapu and PTHA18 magnitude-frequency models on the Kermadec-Tonga thrust source. (a) The Kermadec-Tonga thrust source-zone as represented in PTHA18, with key sites featuring in this study. (b) The island of Tongatapu, with four offshore sites from PTHA18 that are used in this study. (c) The unsegmented magnitude–frequency model from PTHA18, which is given 50 per cent weight. Light-grey regions depict 32 000 alternative magnitude–frequency models that represent epistemic uncertainties in scenario-frequencies. Data show thrust-like earthquakes on the source-zone in 1976–2016, as used in PTHA18 (green triangles). The logic-tree mean and percentile curves summarize the alternative models and their weights. (d–f) The segmented magnitude–frequency models used in PTHA18, which are similar to the unsegmented model but derived using the geometry and data for each segment. The union of these three segments is assigned 50 per cent weight.

To demonstrate Monte Carlo techniques for offshore-to-onshore PTHA, we use offshore earthquake-tsunami scenarios and scenario-frequency models from the 2018 Australia Probabilistic Tsunami Hazard Assessment (henceforth PTHA18, Davies & Griffin 2018, 2020). Although focused on Australia, PTHA18 includes results at globally distributed sites. Herein PTHA18 is used to model earthquake-tsunami inundation hazard in Tongatapu, the main island of Tonga (Figs 1a and b).

For simplicity our calculations are restricted to thrust earthquakes on the full Kermadec-Tonga source-zone (Fig. 1a). This is the dominant source of large earthquake-generated tsunamis near Tongatapu according to the PTHA18 mean scenario-frequency model, producing 90 per cent of tsunamis exceeding 2 m at Site P in Fig. 1(b). Instrumental and historical observations indicate that great (*M*_w_ > 8) tsunamigenic thrust earthquakes have occurred on the Kermadec-Tonga source-zone (e.g. Okal *et al*. 2004; Tang *et al*. 2008; Romano *et al*. 2021) and palaeotsunami deposits are suggestive of older events (Goff *et al*. 2011; Power *et al*. 2012; Lamarche *et al*. 2015; Goff *et al*. 2020). However, Tongatapu is also exposed to other tsunami sources including far-field earthquakes (Davies & Griffin 2020), outer-rise earthquakes (Lay *et al*. 2010), more complex local earthquakes (Okal *et al*. 2011), landslides and volcanoes (Frohlich *et al*. 2009; Goff 2011; Lavigne *et al*. 2021; Duncombe 2022). These are not treated herein and so our hazard results are incomplete, albeit sufficient for our primary purpose of illustrating Monte Carlo techniques for offshore-to-onshore PTHA.

PTHA18 represents the Kermadec-Tonga thrust source-zone using a set of pure-thrust unit-sources (Fig. 1a) with geometry based on SLAB2.0 (Hayes *et al*. 2018). Earthquake scenarios are represented as linear combinations of unit-sources, with magnitudes ranging from 7.2 to 9.6 in increments of 0.1. Although a range of uniform/heterogeneous slip earthquake models are included in PTHA18 and seem consistent with DART buoy observations (Davies 2019; Davies & Griffin 2020), the hazard modelling herein exclusively uses heterogeneous-slip scenarios with constant rigidity. This is because those scenarios performed well in testing (Davies 2019) and because many studies emphasize the importance of slip-heterogeneity in near-field settings (e.g. Geist 2002; Mueller *et al*. 2015; Melgar *et al*. 2019), although we note that compact uniform-slip scenarios may also perform well by using an artificially small rupture area and correspondingly high slip (An *et al*. 2018; Davies 2019).

On the Kermadec-Tonga trench, PTHA18 represents earthquake scenario-frequencies using an equally weighted combination of unsegmented and segmented representations. Three segments are distinguished for the latter; Tonga, Kermadec, and Hikurangi (Fig. 1a), and their combination is termed the ‘union-of-segments’. The segments do not represent hard-boundaries (i.e. earthquake scenarios can rupture on multiple segments), but represent the possibility that key source-zone parameters vary on each segment (e.g. maximum-magnitude, coupling, *b*-value) which the unsegmented model cannot. A family of 32 000 magnitude-frequency models are associated with each of the three segments and the unsegmented model (Figs 1c–f, details in Davies & Griffin 2020). The magnitude-frequency models are defined using a logic-tree and weighted using Bayesian techniques that leverage earthquake catalogue data, plate-convergence rates, and uncertain prior information on maximum magnitudes, coupling and the Gutenberg–Richter *b* value (Davies & Griffin 2020). Each magnitude–frequency curve has an associated scenario-frequency model which partitions the seismicity among scenarios. This partition is non-uniform, even for scenarios with identical magnitudes, to obtain consistency with spatially variable plate convergence and adjust for model biases inferred by comparison with deep-ocean tsunamis (Davies & Griffin 2020).

The PTHA18 results imply large uncertainties in the frequency of high-magnitude earthquakes on the Kermadec-Tonga trench [Figs 1c–f; see Davies & Griffin (2020) for further details]. This reflects the combination of a large area subduction interface (Hayes *et al*. 2018), high plate convergence rates (Bird 2003), uncertain seismic coupling (Berryman *et al*. 2015), and a limited history of large subduction earthquakes (Okal *et al*. 2004, 2011). One interpretation is that there is relatively low coupling of the plate interface (Scholz & Campos 2012) and low maximum-magnitudes (Heuret *et al*. 2012; Bletery *et al*. 2016), in which case large tsunamigenic earthquakes may be relatively infrequent. However the earthquake history is far too short to preclude the possibility of earthquakes much larger than historically observed (McCaffrey 2008; Zöller 2013; Rong *et al*. 2014; Zöller 2017). Such events have been suggested on the basis of palaeotsunami evidence (Power *et al*. 2012; Goff *et al*. 2020) and seem plausible given the fault geometry (McCaffrey 2008; Davies *et al*. 2017; Kalligeris *et al*. 2017; Power *et al*. 2017; Plescia & Hayes 2020). As a result there is much uncertainty as to the potential for large earthquakes on the Kermadec-Tonga trench (Fig. 1; Berryman *et al*. 2015; UNESCO/IOC 2020). This is reflected in the family of scenario-frequency models defined by PTHA18 and used herein.

## 3 MONTE CARLO INTEGRATION TECHNIQUES FOR OFFSHORE-TO-ONSHORE PTHA

### 3.1 Synthetic catalogues

In PTHA it is common for exceedance-rate curves (eq. 1) to be approximated at coastal sites by sampling a random synthetic catalogue with very long duration *D* (years) from the offshore PTHA (e.g. Lane *et al*. 2012; Hoechner *et al*. 2016; Li *et al*. 2016b, a; Power *et al*. 2017; Li *et al*. 2018). Below we analyse the Monte Carlo accuracy of synthetic catalogues.

Denoting the set of scenarios in the synthetic catalogue as *E*^SC^, exceedance-rates are estimated as:

(2) $$\begin{equation*}
\widehat{\lambda _{i}^{\rm SC}}\left(Q \gt Q^{T} \right) = \frac{1}{D} \left ( \sum _{e \in E^{\rm SC}} \mathbb {1}_{\left(Q(e) \gt Q^{T}\right)} \right ) .
\end{equation*}
$$

Here the hat notation $\widehat{\lambda _{i}^{\rm SC}}(Q\gt Q^T)$ implies this is an *estimate* of the value of *λ_i_*(*Q* > *Q*^*T*^) which is defined by eq. (1); the error in eq. (2) varies for each random synthetic catalogue. The term in large parenthesis on the right hand side is a random integer with mean [*Dλ_i_*(*Q* > *Q*^*T*^)] and has a Poisson distribution in the common case that the synthetic catalogue is temporally distributed as a homogeneous Poisson process, with independent and identically distributed scenarios (Luen & Stark 2012). Noting the mean and variance of a Poisson random variable are equal (e.g. Bolker 2008) it follows that the average of $\widehat{\lambda _{i}^{\rm SC}}(Q \gt Q^T)$ over all synthetic catalogues equals eq. (1), and their variance *σ*^2^ is:

(3) $$\begin{equation*}
\sigma ^{2} \Big {(} \widehat{\lambda _{i}^{\rm SC}}\left(Q\gt Q^T \right) \Big {)} = \lambda _{i}\left(Q \gt Q^T \right)/D
\end{equation*}
$$

which is the square of the standard deviation of the Monte Carlo errors.

These results enable Monte Carlo errors in eq. (2) to be constrained prior to sampling, which can help modellers determine a suitable catalogue duration. Suppose a synthetic catalogue with duration *D* = 100 000 yr used, and satisfies the assumptions above (e.g. Lane *et al*. 2012; Li *et al*. 2016b, 2018; Power *et al*. 2017). If eq. (1) gives an exceedance-rate of 1/1000, then 95 per cent of Monte Carlo estimates will be within the interval (1/1235, 1/833) derived using a Poisson distribution, or approximately ±20 per cent (using eqs 2–3 and assuming a 2-standard-deviation interval). The typical error increases for rare events; if eq. (1) gives an exceedance-rate of 1/10 000, then 95 per cent of Monte Carlo estimates will be in (1/25 000, 1/5882), or approximately ±63 per cent. The acceptability of these errors will vary from case-to-case; while they should be minimized for numerical accuracy, it may be practically acceptable if they are small relative to epistemic uncertainties in the exceedance-rates (Li *et al*. 2016b; Power *et al*. 2017).

Synthetic catalogues are often inefficient for tsunami inundation hazard calculations (Sepúlveda *et al*. 2019; Williamson *et al*. 2020). If seismicity follows a Gutenberg–Richter like distribution then they tend to be dominated by smaller magnitudes, while larger magnitude scenarios are resolved less well despite often being more relevant in tsunami hazard applications. However if only large scenarios are of interest, this can be partly mitigated by ignoring smaller scenarios (Lane *et al*. 2012).

Another inefficiency arises if multiple scenario-frequency models *i* ∈ *I* are used to represent epistemic uncertainties. Because the scenario-frequency is represented by the catalogue itself, in general different synthetic catalogues are required to represent each model *i* ∈ *I* [or at least a sufficiently large random sample of scenario-frequency models should be used, e.g. Power *et al*. (2017)]. This leads to multiplicative growth in the number of scenarios, and simulating these through to inundation may be prohibitively expensive.

### 3.2 Stratified-sampling by magnitude

#### 3.2.1 Sampling approach and statistical properties of the Monte Carlo error

To improve on the efficiency of synthetic catalogues, PTHAs often use stratified-sampling by earthquake magnitude (e.g. De Risi & Goda 2017; Williamson *et al*. 2020; Basili *et al*. 2021; Zamora *et al*. 2021). The Monte Carlo accuracy of this approach is analysed below, and techniques are presented to estimate the errors both before and after high-resolution tsunami simulation.

For stratified-sampling the PTHA scenarios are grouped into discrete magnitude-bins *M*_w,*b*_. This study uses uniformly spaced bins corresponding to those provided in our offshore PTHA (PTHA18):

(4) $$\begin{equation*}
M_{{\rm w},b} \in \Big {\lbrace } (7.15,7.25], (7.25, 7.35], \ldots , (9.55, 9.65] \Big {\rbrace } .
\end{equation*}
$$

On some scenario-frequency models *i* ∈ *I* the higher magnitude bins will be impossible (zero occurrence-rate) depending on the maximum-magnitude for model *i* (details in Davies & Griffin 2020).

From the offshore PTHA, the occurrence-rate of scenarios in magnitude-bin *M*_w,*b*_ is known:

(5) $$\begin{equation*}
\lambda _{i}(M_{{\rm w},b}) = \sum _{e \in E_{b}} r_{i}(e) .
\end{equation*}
$$

Here *E_b_* is the set of all PTHA scenarios in magnitude-bin *M*_w,*b*_. To approximate the exceedance-rate (eq. 1) a number *N*(*M*_w,*b*_) of scenarios are sampled from each *E_b_* using weighted random sampling with replacement. For stratified-sampling, the sampling weights $w_{b,i}^{SS}(e)$ must equal the within-magnitude-bin scenario conditional probability:

(6) $$\begin{equation*}
w_{b,i}^{SS}(e) = \frac{ r_{i}(e) }{\sum _{e \in E_{b}} r_{i}(e) }
\end{equation*}
$$

assuming *λ_i_*(*M*_w,*b*_) > 0 (otherwise no sampling is required). Note the novel sampling scheme proposed in Section 3.3 provides a more flexible analogue of eq. (6).

Denote the set of randomly sampled scenarios in magnitude-bin *M*_w,*b*_ as $E_{b,i}^{SS}$. Suppose the tsunami has been simulated for each. The within-magnitude-bin exceedance-rate may be estimated as:

(7) $$\begin{equation*}
\widehat{\lambda _{i}^{SS}}(Q \gt Q^{T} | M_{{\rm w},b}) = \lambda _{i}(M_{{\rm w},b}) \left ( \sum _{e \in E_{b,i}^{SS}} \mathbb {1}_{\left(Q(e) \gt Q^{T}\right)} \right ) / N(M_{{\rm w},b}) .
\end{equation*}
$$

This varies with the random sample $E_{b,i}^{SS}$. The term in large parenthesis in eq. (7) behaves like a binomial random variable with size *N*(*M*_w,*b*_) and probability *p*_*b,i,T*_, where:

(8) $$\begin{equation*}
p_{b,i,T} = \frac{ \sum _{e \in E_{b}} r_{i}(e) \mathbb {1}_{(Q(e) \gt Q^{T})} }{\sum _{e \in E_{b}} r_{i}(e) }
\end{equation*}
$$

is the probability that a random PTHA scenario in *E_b_* exceeds the threshold *Q*^*T*^. Using standard formulae for the mean and variance of a binomial random variable (Supplementary Material, Section A) it follows that eq. (7) gives an unbiased estimate of the within-magnitude-bin exceedance-rate and has variance:

(9) $$\begin{equation*}
\sigma ^{2} \Big {(} \widehat{\lambda _{i}^{SS}}(Q \gt Q^{T} | M_{{\rm w},b}) \Big {)} = \frac{1}{N(M_{{\rm w},b})} \big {(} \lambda _{i}(M_{{\rm w},b}) \big {)}^{2} p_{b,i,T} (1-p_{b,i,T}) .
\end{equation*}
$$

Calculation of the Monte Carlo variance (eq. 9) requires that *Q*(*e*) is known for all *e* ∈ *E_b_*, to evaluate *p*_*b,i,T*_ in eq. (8). This is possible at offshore sites (using the offshore PTHA) but generally impossible at coastal sites. However an *estimate* of the variance at coastal sites can be derived after the tsunami has been simulated for all random scenarios in $E_{b,i}^{SS}$, via an estimate of *p*_*b,i,T*_ (denoted $\widehat{p}_{b,i,T}$).

(10) $$\begin{eqnarray*}
\widehat{p}_{b,i,T} &=& \left ( \sum _{e \in E_{b,i}^{SS}} \mathbb {1}_{\left(Q(e) \gt Q^{T}\right)} \right )/{N(M_{{\rm w},b})} \nonumber \\
\widehat{\sigma ^{2}} \Big {(} \widehat{\lambda _{i}^{SS}}(Q \gt Q^{T} | M_{{\rm w},b}) \Big {)} &=& \frac{1}{N(M_{{\rm w},b})} \big {(} \lambda _{i}(M_{{\rm w},b}) \big {)}^{2} \widehat{p}_{b,i,T}(1-\widehat{p}_{b,i,T}) .
\end{eqnarray*}
$$

See Supplementary Material Section A for further justification of eqs (9) and (10).

Summation of eq. (7) over magnitude-bins leads to an unbiased estimate of the exceedance-rate (eq. 1) which is of primary interest for PTHA:

(11) $$\begin{equation*}
\widehat{\lambda _{i}^{SS}}\left(Q \gt Q^{T} \right) = \sum _{M_{{\rm w},b} \in \textrm {magnitude-bins}} \widehat{\lambda _{i}^{SS}}(Q \gt Q^{T} | M_{{\rm w},b}) .
\end{equation*}
$$

This will vary for different Monte Carlo samples. Because random samples in different magnitude-bins are independent, the variance of eq. (11) is the sum of the within-magnitude-bin variances (eq. 9):

(12) $$\begin{equation*}
\sigma ^{2} \Big {(} \widehat{\lambda _{i}^{SS}}\left(Q \gt Q^{T} \right) \Big {)} = \sum _{M_{{\rm w},b} \in \textrm {magnitude-bins}} \sigma ^{2} \Big {(} \widehat{\lambda _{i}^{SS}}(Q \gt Q^{T} | M_{{\rm w},b}) \Big {)} .
\end{equation*}
$$

Eq. (12) is termed the *analytical* Monte Carlo variance herein because it is computed without Monte Carlo sampling (at sites where the offshore PTHA is valid). This offers a useful check on stratified-sampling schemes in coastal hazard applications, prior to sampling and running expensive tsunami models. For example if the analytical standard-deviation (i.e. square-root of eq. 12) is a high-fraction of the exceedance-rate (eq. 1) at an offshore site, then we expect nearby coastal-hazard estimates will also be inaccurate and may decide to revise the sampling scheme. Although the Monte Carlo variance can also be computed empirically (from the variance of repeated Monte Carlo samples), eq. (12) is faster and simpler. It also plays a core role in the design of non-uniform sampling strategies (Section 3.4).

The analytical Monte Carlo variance (eq. 12) cannot be computed at coastal sites because *Q*(*e*) is not known for all *e* ∈ *E*. However it can be *estimated* after scenarios are sampled and inundation simulated, by summation over eq. (10):

(13) $$\begin{equation*}
\widehat{\sigma ^{2}} \Big {(} \widehat{\lambda _{i}^{SS}}\left(Q \gt Q^{T} \right) \Big {)} = \sum _{M_{{\rm w},b} \in \textrm {magnitude-bins}} \widehat{\sigma ^{2}} \Big {(} \widehat{\lambda _{i}^{SS}}(Q \gt Q^{T} | M_{{\rm w},b}) \Big {)} .
\end{equation*}
$$

This *single-Monte Carlo-sample* variance estimate is useful at coastal sites, where in practice *Q*(*e*) is only known for the random scenarios. Eq. (13) enables the numerical reliability of the Monte Carlo exceedance-rate to be estimated, without additional simulations.

To test this Monte Carlo approach we consider the tsunami maximum-stage exceedance-rates at Site P, located in deep-water east of Tongatapu (Fig. 1b). Only thrust earthquakes on the Kermadec-Tonga source-zone are considered. At Site P the exceedance-rates are defined by the offshore PTHA (eq. 1) and can be evaluated without Monte Carlo sampling; this enables the Monte Carlo estimates to be tested against known values. Scenario rates *r_i_*(*e*) were determined from the logic-tree mean scenario-frequency model $\overline{r}(e)$ in PTHA18, which has 50:50 weight on the unsegmented and union-of-segments models (Fig. 1). Epistemic uncertainties in scenario-frequencies are ignored for simplicity (but considered later).

**Figure 2.**
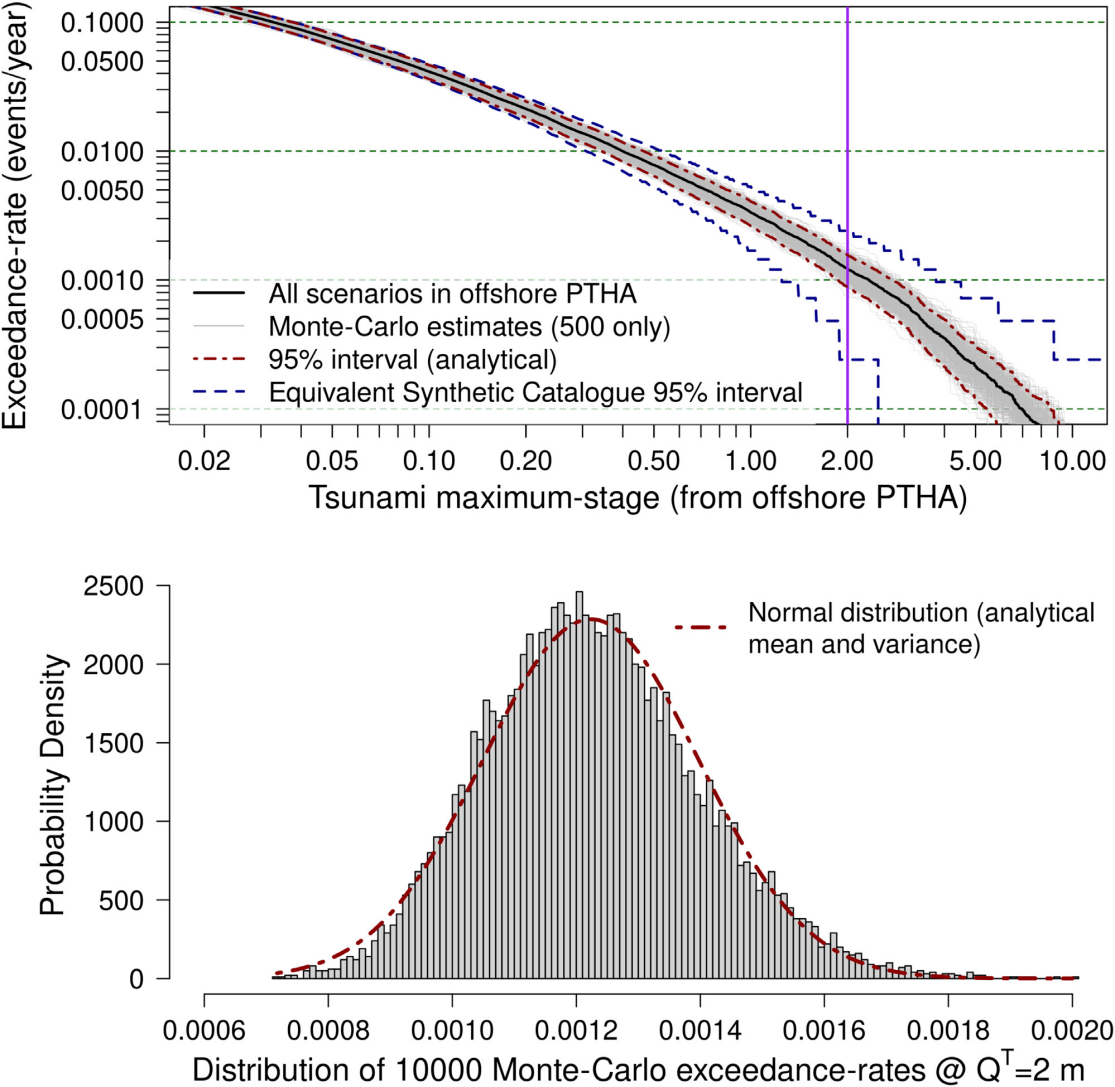
Performance of stratified-sampling at offshore Site P (location in Fig. 1) for PTHA18 thrust scenarios on the Kermadec-Tonga trench. Top panel: logic-tree mean exceedance-rate curve and Monte Carlo estimates. Grey curves depict 500 of the 10 000 Monte Carlo estimates. Bottom panel: histogram of the 10 000 Monte Carlo exceedance-rates for *Q*^*T*^ = 2 m, compared with a normal distribution having mean and variance derived from eqs (1) and (12) respectively.

Ten-thousand Monte Carlo exceedance-rate curves were computed at Site P (Fig. 2). Each Monte Carlo sample uses 1200 random scenarios, with *N*(*M*_w_) = 48 in each magnitude-bin (eq. 4). This corresponds to a computationally feasible number of inundation simulations for our case-study (Section 4) and is constrained by model run-times and computational resources.

The variability of the Monte Carlo exceedance-rates is visually clear (Fig. 2). To compare this stratified-sampling technique with synthetic catalogues, the equivalent synthetic catalogue duration *D* was defined such that 1200 random scenarios are included on average (implying an equivalent computational effort for inundation calculations). Comparison of 95 per cent intervals for the synthetic catalogue and stratified-sampling confirms that errors in the latter grow more slowly at rare return periods, which shows the benefit of magnitude stratification (Fig. 2).

To highlight the validity of the Monte Carlo technique and the *analytical* and *single-Monte Carlo-sample* variance calculations, consider the stage-threshold *Q*^*T*^ = 2 m (Fig. 2). At this threshold the exceedance-rate in eq. (1) differs by only 0.2 per cent from the average of the Monte Carlo estimates computed with eq. (11). Furthermore the empirical standard-deviation of the 10 000 Monte Carlo estimates differs by only 0.3 per cent from the analytical standard-deviation (square root of eq. 12). The distribution of the Monte Carlo results can be approximated with a normal distribution having mean and variance equal to eqs (1) and (12), both of which are computed without Monte Carlo sampling (Fig. 2). This was used to compute the 95 per cent confidence-intervals in Fig. 2. The key point is that, at offshore sites, we can quantify the performance of stratified-sampling before conducting Monte Carlo simulation. This enables the sampling scheme performance to be checked and perhaps further optimized, prior to expensive tsunami simulation.

At coastal sites the offshore PTHA cannot accurately represent the tsunami, so *Q*(*e*) is only known for a single Monte Carlo sample (1200 scenarios) after tsunami simulations are completed. In that case eqs (11) and (13) provide a *single-Monte Carlo-sample* estimate of the exceedance-rate and its Monte Carlo variance, and an approximate 95 per cent confidence-interval for the exceedance-rate in eq. (1) can be constructed by substitution into a normal distribution:

(14) $$\begin{equation*}
\widehat{\lambda _{i}^{SS}}\left(Q \gt Q^{T} \right) \pm 1.96 \sqrt{ \widehat{\sigma ^{2}} \Big {(} \widehat{\lambda _{i}^{SS}}\left(Q \gt Q^{T} \right) \Big {)} } .
\end{equation*}
$$

If Monte Carlo sampling is repeated then this interval will cover the exceedance-rate in eq. (1) approximately, but not exactly, 95 per cent of the time. For the 10 000 Monte Carlo samples in Fig. 2, the empirical coverage was 92.65 per cent at *Q*^*T*^ = 2 m. While approximate, this gives a reasonable indication of the Monte Carlo error at coastal sites when only a single Monte Carlo sample is available.

#### 3.2.2 Stratified-sampling with epistemic uncertainties

How efficient is stratified-sampling when epistemic uncertainties are represented with a family of scenario-frequency models *i* ∈ *I*? Recall synthetic catalogues are inefficient in this context because the scenario-frequencies are represented by the catalogue itself, so a new catalogue is required for each *i* ∈ *I* (Section 3.1). In contrast, for stratified-sampling it is the within-magnitude-bin scenario conditional probabilities that are represented by the catalogue itself (via eq. 6). Thus a new stratified-sample should be constructed for each variation of eq. (6) among the scenario-frequency models *i* ∈ *I*.

Fortunately, in practice PTHA methodologies often assume the within-magnitude-bin scenario conditional probabilities are independent of the particular scenario-frequency model, which facilitates the reuse of stratified-samples. This holds for PTHAs that assume all scenarios within a magnitude-bin are equally likely (e.g. Lorito *et al*. 2015; Williamson *et al*. 2020). It also holds for the approach of Davies & Griffin (2020) which uses non-uniform conditional probabilities to represent spatial variations in tectonics and correct for earthquake model biases. For PTHA18, the within-magnitude-bin scenario conditional probabilities are identical for all 32 000 scenario-frequency models within the unsegmented model (Fig. 1c), and separately, within each of the three segments (Figs 1d–f). These four source models share the same scenarios but with different within-magnitude-bin scenario conditional probabilities (full details in Davies & Griffin 2018, 2020). Scenario sharing is possible because, for example, a scenario on the Tonga segment can also occur on the unsegmented model, and a rupture crossing segment boundaries receives a partial occurrence-rate from each included segment. However a scenario that is fully contained in one segment will have zero occurrence-rate on the other segments. Thus the within-magnitude-bin scenario conditional probabilities must vary between segments. To represent this with stratified-sampling, four different versions of eq. (6) must be considered: Unsegmented, Tonga segment, Kermadec segment and Hikurangi segment.

Thus for this study, epistemic uncertainty calculations via stratified-sampling require four sets of random scenarios. Each corresponds to a different panel in Figs 1(c)–(f), and the 1200 scenarios must be partitioned among these; within each the Monte Carlo sample can be reused for every scenario-frequency model. The use of four sets of scenarios represents a big improvement over synthetic catalogues, which would require a separate catalogue for every *i* ∈ *I*, or at least a large random sample of those models (Power *et al*. 2017).

### 3.3 Improving the efficiency near a site of interest: stratified/importance-sampling informed by offshore wave heights

#### 3.3.1 Sampling approach and statistical properties of the Monte Carlo error

This section develops a novel ‘stratified/importance-sampling’ scheme for offshore-to-onshore PTHA which is more efficient than stratified-sampling near a specified site of interest, but potentially less efficient far from that site. As with stratified-sampling, techniques are presented to estimate the Monte Carlo errors both before and after tsunami inundation simulation.

Our site of interest is exposed to a large earthquake source-zone (Figs 1a and b) and for scenarios within a magnitude-bin, the tsunami impacts will vary greatly with the earthquake location and slip-distribution. Suppose a rough estimate of the ‘scenario importance’ for our site can be derived from the offshore PTHA, without inundation computation. Improved Monte Carlo exceedance-rates might be obtained by preferentially sampling more important scenarios, with appropriate weighting to prevent bias. The theory of importance-sampling provides a framework for achieving this, with variants of the approach used in many fields (e.g. Owen & Zhou 2000; Tokdar & Kass 2009; Lie & Quer 2017). The approach herein is termed ‘stratified/importance-sampling’ because scenarios are also stratified by magnitude.

The method requires a user-defined notion of the ‘scenario importance’. This is a positive number $\mathcal {I}(e)$ associated with every scenario *e* ∈ *E*; in each magnitude-bin the chance of randomly sampling a scenario will be rescaled in proportion to $\mathcal {I}(e)$. This study uses the PTHA18 maximum-stage at Site P (Fig. 1b):

(15) $$\begin{equation*}
\mathcal {I}(e) = \textrm {PTHA18~maximum-stage~at~point~} P \textrm {~for~scenario~} e
\end{equation*}
$$

Alternative definitions of $\mathcal {I}(e)$ can be used; good choices should be indicative of inundation potential at the site of interest, easy to compute, and avoid reference to a specific scenario-frequency model. Depending on the site, factors such as the peak-to-trough wave height, wave spectra or directionality (Power & Tolkova 2013) could be useful for defining *I*(*e*). A good choice of *I*(*e*) will make the method more computationally efficient, while a poor choice could reduce the accuracy compared with stratified-sampling. As discussed below, the latter risk can be mitigated by testing the approach at nearby offshore sites (before inundation computation), and by estimating the errors at onshore sites (after inundation computation).

Suppose that scenarios are grouped into discrete magnitude-bins *M*_w,*b*_, and a user-specified number *N*(*M*_w,*b*_) are sampled per-bin using weighted random sampling with replacement. In contrast to stratified-sampling, the chance $w_{b,i}^{\rm SIS}(e)$ of sampling each scenario is:

(16) $$\begin{equation*}
w_{b,i}^{\rm SIS}(e) = \frac{\mathcal {I}(e) r_{*}(e) }{\sum _{e \in E_b} \mathcal {I}(e) r_{*}(e) } .
\end{equation*}
$$

This is identical to standard stratified-sampling if $\mathcal {I}(e)$ is constant and r_*_(*e*) = *r_i_*(*e*) (eq. 6). If only one model *i* ∈ *I* is considered then the function r_*_(*e*) should equal *r_i_*(*e*), but other choices are useful when treating epistemic uncertainties in scenario-frequencies (Section 3.3.2). Any choice of r_*_(*e*) should satisfy r_*_(*e*) > 0 whenever *r_i_*(*e*) > 0, to retain a non-zero chance of sampling scenario *e*.

Using the random scenarios in magnitude-bin *M_w,b_* (denoted $E_{b,i}^{\rm SIS}$) the within-magnitude-bin exceedance-rate is estimated as:

(17) $$\begin{equation*}
\widehat{\lambda _{i}^{\rm SIS}}(Q \gt Q^{T} | M_{{\rm w},b}) = \lambda _{i}(M_{{\rm w},b}) \left ( \sum _{e \in E_{b,i}^{\rm SIS}} \phi _{b,i}^{\rm SIS}(e) \mathbb {1}_{(Q(e) \gt Q^{T})} \right )/N(M_{{\rm w},b}) .
\end{equation*}
$$

This is similar to stratified-sampling (eq. 7) except importance-sampling weights $\phi _{b,i}^{\rm SIS}(e)$ appear, to correct for the biased sampling. Using the approach of ‘basic importance-sampling’ (e.g. Owen & Zhou 2000; Tokdar & Kass 2009) these weights are:

(18) $$\begin{equation*}
\phi _{b,i}^{\rm SIS}(e) = \left ( \frac{ r_{i}(e) }{\sum _{e \in E_b} r_{i}(e) } \right )/ w_{b,i}^{\rm SIS}(e)
\end{equation*}
$$

For impossible scenarios (r_*_(*e*) = 0) eq. (18) involves zero division so is undefined; these cases are set zero.

Basic importance-sampling is unbiased (e.g. Owen & Zhou 2000; Tokdar & Kass 2009) thus eq. (17) is on average equal to the within-magnitude-bin exceedance-rate. It varies with the random sample $E_{b,i}^{\rm SIS}$ and is asymptotically normal as *N*(*M*_w,*b*_) → ∞, with variance (Geweke 1989; Tokdar & Kass 2009):

(19) $$\begin{equation*}
\sigma ^{2} \Big {(} \widehat{\lambda _{i}^{\rm SIS}}(Q \gt Q^{T} | M_{{\rm w},b}) \Big {)} = \frac{\Big {(}\lambda _{i}(M_{{\rm w},b})\Big {)}^{2}}{N(M_{{\rm w},b})} \sum _{e \in E_b} \Big {(} \big {[} \mathbb {1}_{(Q(e) \gt Q^{T})} \phi _{b,i}^{\rm SIS}(e) - p_{b,i,T} \big {]}^2 w_{b,i}^{\rm SIS}(e) \Big {)}
\end{equation*}
$$

Eq. (19) is proved in the Supplementary Material, Section B. It is analogous to eq. (9) for stratified-sampling, and is identical if $w_{b,i}^{SS}(e) = w_{b,i}^{\rm SIS}(e)$. In both cases the analytical variance computation does not require any Monte Carlo sampling, but does require that *Q*(*e*) is known for all scenarios in *E_b_* which prevents its use at coastal sites (where the offshore PTHA does not accurately model the tsunami). But as with stratified-sampling, an estimate of the variance can be derived using a single Monte Carlo sample (Tokdar & Kass 2009):

(20) $$\begin{equation*}
\widehat{\sigma ^{2}} \Big {(} \widehat{\lambda _{i}^{\rm SIS}}(Q \gt Q^{T} | M_{{\rm w},b}) \Big {)} = \frac{\Big {(}\lambda _{i}(M_{{\rm w},b})\Big {)}^{2}}{N(M_{{\rm w},b})} \sum _{e \in E_{b,i}^{\rm SIS}} \Big {(} [ \mathbb {1}_{(Q(e) \gt Q^{T})} \phi _{b,i}^{\rm SIS}(e) - \widehat{q}_{b,i,T} ]^{2}/N(M_{{\rm w},b}) \Big {)} ,
\end{equation*}
$$

where

(21) $$\begin{equation*}
\widehat{q}_{b,i,T} = \left ( \sum _{e \in E_{b,i}^{\rm SIS}} \phi _{b,i}^{\rm SIS}(e) \mathbb {1}_{(Q(e) \gt Q^{T})} \right )/N(M_{{\rm w},b})
\end{equation*}
$$

is an estimate of *p*_*b,i,T*_ (eq. 8). See Supplementary Material Section B for further justification of eqs (19) and (20).

Analogues of key equations for stratified-sampling (eqs 11–13) follow. Summation of eq. (17) over magnitude-bins gives an unbiased estimate of the exceedance-rate in eq. (1):

(22) $$\begin{equation*}
\widehat{\lambda _{i}^{\rm SIS}}\left(Q \gt Q^{T} \right) = \sum _{M_{{\rm w},b} \in \textrm {Magnitude~bins}} \widehat{\lambda _{i}^{\rm SIS}}(Q \gt Q^{T} | M_{{\rm w},b})
\end{equation*}
$$

The *analytical* variance of this estimate is the sum of the within magnitude-bin variances (eq. 19):

(23) $$\begin{equation*}
\sigma ^{2} \Big {(} \widehat{\lambda _{i}^{\rm SIS}}\left(Q \gt Q^{T} \right) \Big {)} = \sum _{M_{{\rm w},b} \in \textrm {magnitude-bins}} \sigma ^{2} \Big {(} \widehat{\lambda _{i}^{\rm SIS}}(Q \gt Q^{T} | M_{{\rm w},b}) \Big {)}
\end{equation*}
$$

Eq. (23) enables the Monte Carlo exceedance-rate variance to be calculated prior to sampling (at offshore sites), facilitating checks of the sampling scheme prior to inundation simulation.

Although eq. (23) cannot generally be computed at coastal sites, the variance can be estimated when tsunami simulations are completed for a *single-Monte Carlo-sample* by summation over eq. (20):

(24) $$\begin{equation*}
\widehat{\sigma ^{2}} \Big {(} \widehat{\lambda _{i}^{\rm SIS}}\left(Q \gt Q^{T} \right) \Big {)} = \sum _{M_{{\rm w},b} \in \textrm {magnitude-bins}} \widehat{\sigma ^{2}} \Big {(} \widehat{\lambda _{i}^{\rm SIS}}(Q \gt Q^{T} | M_{{\rm w},b}) \Big {)}
\end{equation*}
$$

**Figure 3.**
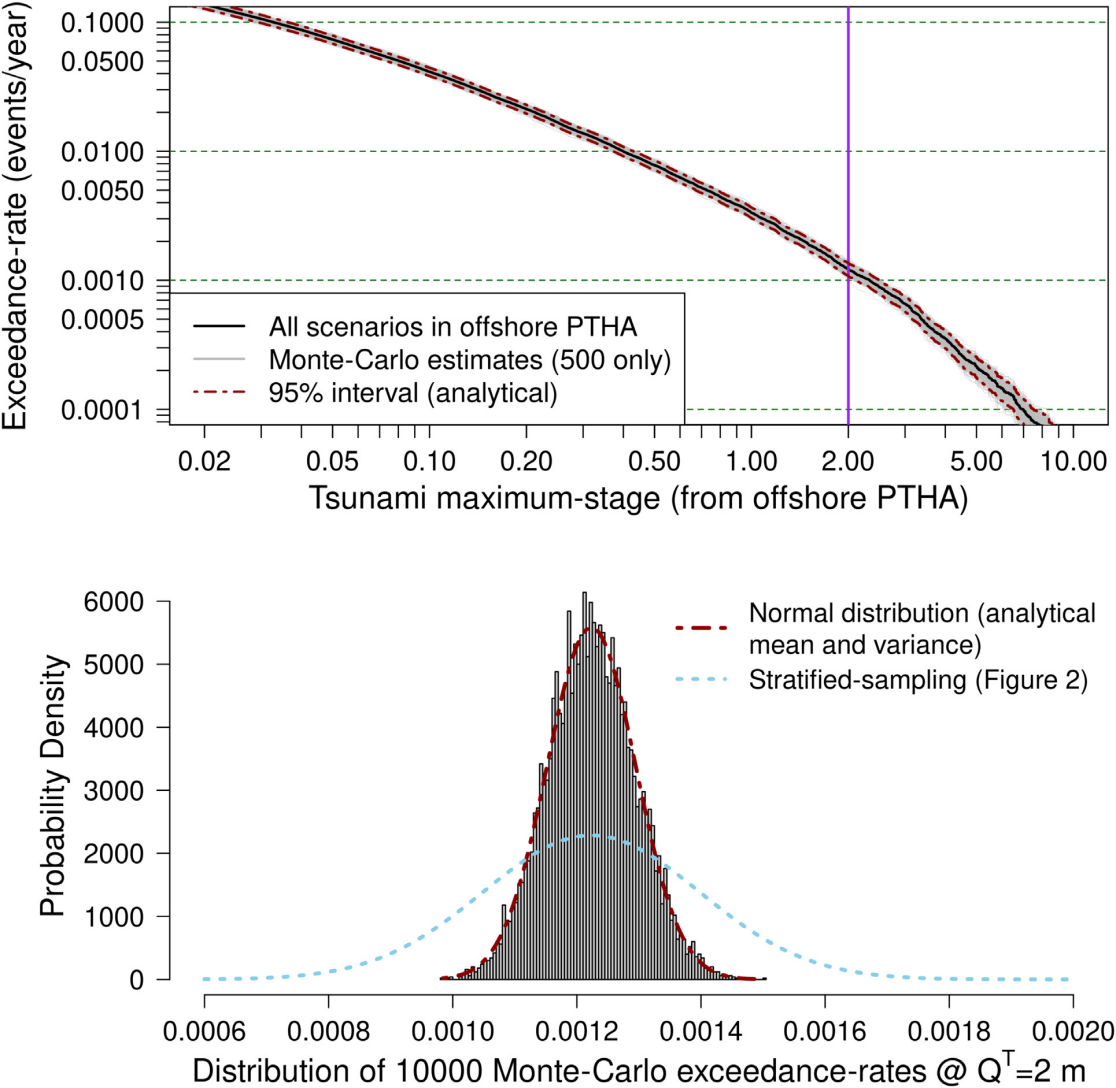
Performance of stratified/importance-sampling at an offshore Site P (location in Fig. 1) for PTHA18 thrust scenarios on the Kermadec-Tonga trench. Top panel: logic-tree mean exceedance-rate curve and Monte Carlo estimates. Grey curves depict 500 of the 10 000 Monte Carlo. Bottom panel: histogram of the 10 000 Monte Carlo exceedance-rates for *Q*^*T*^ = 2 m, compared with a normal distribution having mean and variance derived from eqs (1) and (23), respectively. For comparison we show the normal distribution from stratified-sampling (see bottom-panel of Fig. 2).

To demonstrate the improved accuracy, Fig. 3 repeats the Monte Carlo test at Site P using stratified/importance-sampling. Because the previous stratified-sampling results apply to the logic-tree mean scenario frequency model $\overline{r}(e)$, for stratified/importance-sampling we set $r_{*}(e) = \overline{r}(e)$ in eq. (16). Compared with stratified-sampling (Fig. 2) the reduction in the Monte Carlo error is visually obvious; for example at *Q*^*T*^ = 2 m the empirical standard deviation of the Monte Carlo exceedance-rates is 2.45 times smaller. To achieve similar accuracy with stratified-sampling would require using 2.45^2^ = 6 times as many scenarios (7200 versus 1200) and thus a factor of 6 increase in the computational effort.

In this example the average of the 10 000 Monte Carlo exceedance-rates at *Q*^*T*^ = 2 m differs from eq. (1) by only 0.05 per cent, reflecting the lack of bias. The analytical standard-deviation (square root of eq. 23) agrees with the Monte Carlo standard-deviation within 0.6 per cent, confirming the accuracy of the analytical variance (Fig. 3). Furthermore, a *single-Monte Carlo-sample* approximate confidence interval for the exceedance-rate (eq. 1) can be derived as for eq. (14), which is particularly useful at coastal sites:

(25) $$\begin{equation*}
\widehat{\lambda _{i}^{\rm SIS}}\left(Q \gt Q^{T} \right) \pm 1.96 \sqrt{ \widehat{\sigma ^{2}} \Big {(} \widehat{\lambda _{i}^{\rm SIS}}\left(Q \gt Q^{T} \right) \Big {)} }.
\end{equation*}
$$

For the 10 000 Monte Carlo samples in Fig. 3 this approximate confidence-interval included the eq. (1) exceedance-rate 94.55 per cent of the time, very close to the ideal value of 95 per cent. These results confirm the utility of the *analytical* and *single-Monte Carlo-sample* estimates of the Monte Carlo exceedance-rate variance.

**Figure 4.**
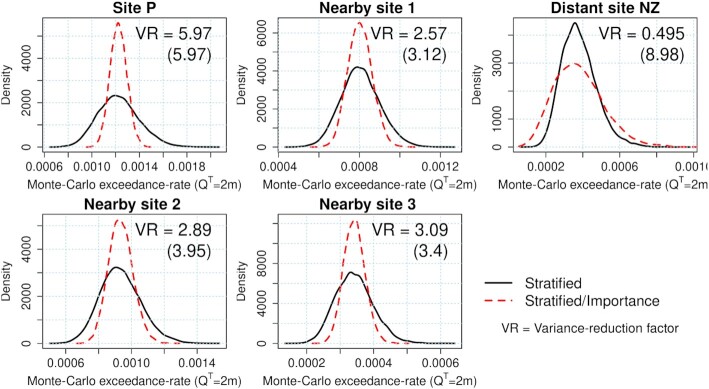
Variability of Monte Carlo exceedance-rate estimates for *Q*^*T*^ = 2 m using stratified and stratified/importance-sampling (10 000 Monte Carlo samples, each with 1200 scenarios). Site locations shown in Fig. 1(a) and (b). VR gives the ratio of the Monte Carlo variances with stratified and stratified/importance-sampling. Site P was used to define the scenario importance in all cases; but numbers in parenthesis show the VR value that would be obtained if the site’s own tsunami-maxima were used instead. All calculations use the logic-tree mean scenario-frequency model.

Crucially, the improved Monte Carlo efficiency is not restricted to Site P that was used to define the scenario importance. If the tsunami is large at Site P it also tends to be large at nearby sites, which also show reductions in the Monte Carlo variance (see nearby sites 1, 2 and 3 in Fig. 4, with locations in Fig. 1b). The ratio of the Monte Carlo variances associated with each sampling method, denoted VR and shown in Fig. 4, is equivalent to the increase in computational effort required to make stratified-sampling as accurate as stratified/importance-sampling. The VR values in Fig. 4 were computed analytically (eq. 12/eq. 23), but very similar values were obtained using empirical variances of the Monte Carlo samples (within 3.1 per cent). At sites near to Site P, VR is comparable to that which would be obtained by using each site’s tsunami maxima to define its own $\mathcal {I}(e)$ (shown in parenthesis in panels of Fig. 4). Efficiency improvements are also expected at the onshore site of interest (Tongatapu) because it is near Site P; this is the core idea behind stratified/importance-sampling.

While performing well near to Site P, the definition of $\mathcal {I}(e)$ will not give a good proxy of the tsunami size far from Site P. While the method remains valid, the Monte Carlo error can increase relative to stratified-sampling. For example this occurs at ‘Distant site NZ’ about 2000 km south of Site P (Fig. 4, see site location in Fig. 1a). If the goal is to conduct a hazard assessment near Site P then the reduced accuracy at distant sites is of little concern. If the goal were to conduct a hazard assessment near ‘Distant site NZ’, then Site P is not a good choice for defining the scenario importance $\mathcal {I}(e)$. For example at ‘Distant site NZ’, a factor 8.98 efficiency improvement could be obtained with a local definition of $\mathcal {I}(e)$ (Fig. 4).

These results suggest stratified/importance-sampling based on Site P will be useful throughout the Tongatapu region. We recommend conducting similar checks in coastal hazard applications, prior to running expensive tsunami simulations. With a poor choice of $\mathcal {I}(e)$ importance-sampling might perform badly (like at Distant Site NZ, Fig. 4) or even fail (e.g. if the tails of the distribution are poorly represented, Owen & Zhou 2000). In the context of PTHA this would likely result in poor performance at nearby offshore sites, which can be detected and fixed at little computational cost.

#### 3.3.2 Stratified/importance sampling with epistemic uncertainties

Can a single Monte Carlo sample obtained with stratified/importance-sampling be re-used for epistemic uncertainty calculations? The key issue is whether the chance of sampling any scenario (eq. 16) is unchanged for multiple scenario-frequency models *i* ∈ *I*. Section 3.2.1 showed that for stratified-sampling, the chance of sampling each scenario (eq. 6) is equal to the within-magnitude-bin scenario conditional probability, necessitating four sets of stratified-samples in the context of this study (one for the unsegmented model, and one for each segment). In contrast stratified/importance-sampling is more flexible by virtue of the scenario weighting (eqs 16 and 18). This enables sharing of Monte Carlo scenarios between the unsegmented model and the segments, and is an important advantage over stratified-sampling.

In our onshore application a single Monte Carlo sample is created by setting r_*_(*e*) equal to the logic-tree mean model $\overline{r}(e)$. The chance of sampling each scenario in magnitude-bin *M*_w,*b*_ (eq. 16) is thus defined as:

(26) $$\begin{equation*}
w_{b,i}^{\rm SIS}(e) = \frac{\mathcal {I}(e) \overline{r}(e) }{\sum _{e \in E_b} \mathcal {I}(e) \overline{r}(e) }
\end{equation*}
$$

which does not change when varying *i* ∈ *I*. With this approach the reasoning behind eqs (17)–(25) remains valid, whether applied to different segment models (Tonga, Kermadec or Hikurangi) or the unsegmented model. Therefore each of these four models can use the same set of 1200 scenarios. This offers a potentially large improvement over stratified-sampling, where the need to partition the 1200 scenarios among the 4 source models would result in lower sample sizes on each.

In practice the benefit of scenario sharing depends on the extent to which different source models assign non-zero probabilities to the same scenarios, in addition to how $\mathcal {I}(e)$ is defined. Thus the performance should be checked in each application at offshore sites, prior to expensive inundation simulation. Here this is deferred to Section 3.5 where we also assess the effect of non-uniform magnitude-bin sampling.

### 3.4 Improving the efficiency: non-uniform sampling of magnitude-bins

This section develops an approach to determine the number of samples in each magnitude-bin *N*(*M*_w,*b*_). Both stratified and stratified/importance-sampling require fixing *N*(*M*_w,*b*_) prior to sampling, and in practice uniform *N*(*M*_w, *b*_) are often used (e.g. De Risi & Goda 2017; Williamson *et al*. 2020; Zamora *et al*. 2021). However the computational effort is generally related to the total number of scenarios *N*_tot_:

(27) $$\begin{equation*}
N_{\rm tot} = \sum _{M_{{\rm w},b} \in \textrm {Magnitude~bins}} N(M_{{\rm w},b})
\end{equation*}
$$

which determines how many high-resolution tsunami simulations are required. Below we develop a strategy to select non-uniform *N*(*M*_w,*b*_) values that reduce the Monte Carlo errors while keeping *N*_tot_ constant. The approach is found to be beneficial, especially for relatively rare large tsunamis.

As a starting point it is shown that, prior to sampling, optimal values of *N*(*M*_w,*b*_) can be computed at any *offshore* site using the offshore PTHA, given a specific threshold *Q*^*T*^ and scenario-frequency model *i* ∈ *I*. The solution is optimal in the sense that it minimizes the variance of the Monte Carlo exceedance-rate estimates. Similar problems and solution strategies are common in sampling theory (e.g. Thompson 2012).

For both stratified and stratified/importance-sampling, the analytical variance of the Monte Carlo exceedance-rate estimate is of the form (eqs 12 and  23):

(28) $$\begin{equation*}
\sum _{M_{{\rm w},b} \in \textrm {Magnitude~bins}} \frac{\alpha _{i}(M_{{\rm w},b} | Q^{T})}{N(M_{{\rm w},b})}
\end{equation*}
$$

where the *α_i_*(*M*_w,*b*_|*Q*^*T*^) are obtained by removing the factor 1/*N*(*M*_w,*b*_) from the within-magnitude-bin variance, that is using eq. (9) for stratified-sampling:

(29) $$\begin{equation*}
\alpha _{i}(M_{{\rm w},b} | Q^{T}) = \big {(} \lambda _{i}(M_{{\rm w},b}) \big {)}^{2} p_{b,i,T} (1-p_{b,i,T})
\end{equation*}
$$

and eq. (19) for stratified/importance-sampling:

(30) $$\begin{equation*}
\alpha _{i}(M_{{\rm w},b} | Q^{T}) = \big {(} \lambda _{i}(M_{{\rm w},b}) \big {)}^{2} \left ( \sum _{e \in E_b} \big {(} \big {[} \mathbb {1}_{(Q(e) \gt Q^{T})} \phi _{b,i}^{\rm SIS}(e) - p_{b,i,T} \big {]}^2 w_{b,i}^{\rm SIS}(e) \big {)} \right )
\end{equation*}
$$

In either case the values of *N*(*M*_w,*b*_) that minimize the variance (eq. 28) subject to the constraint of fixed *N*_tot_ (eq. 27) can be derived using Lagrange multipliers, and are denoted *N_i_*(*M*_w,*b*_|*Q*^*T*^):

(31) $$\begin{equation*}
N_{i}(M_{{\rm w},b} | Q^T) = N_{\rm tot} \sqrt{\alpha _{i}(M_{{\rm w},b} | Q^{T})} \Big {/} \left ( \sum _{M_{{\rm w},b} \in \textrm {Magnitude~bins}} \sqrt{\alpha _{i}(M_{{\rm w},b} | Q^{T})} \right )
\end{equation*}
$$

For practical applications eq. (31) should be rounded to give integer solutions in each magnitude-bin.

The optimal sampling effort (eq. 31) is subject to important limitations; it can only be computed where the offshore PTHA is accurate (i.e. not at coastal sites) and is not necessarily robust for alternative scenario-frequency models *i* ∈ *I*, alternative offshore sites, or alternative thresholds. However we suggest that good sampling strategies (albeit non-optimal) can be inferred by inspecting optimal solutions at offshore sites near the coastal site of interest, combined with judgement about the tsunami wave-heights of most significance to the application.

**Figure 5.**
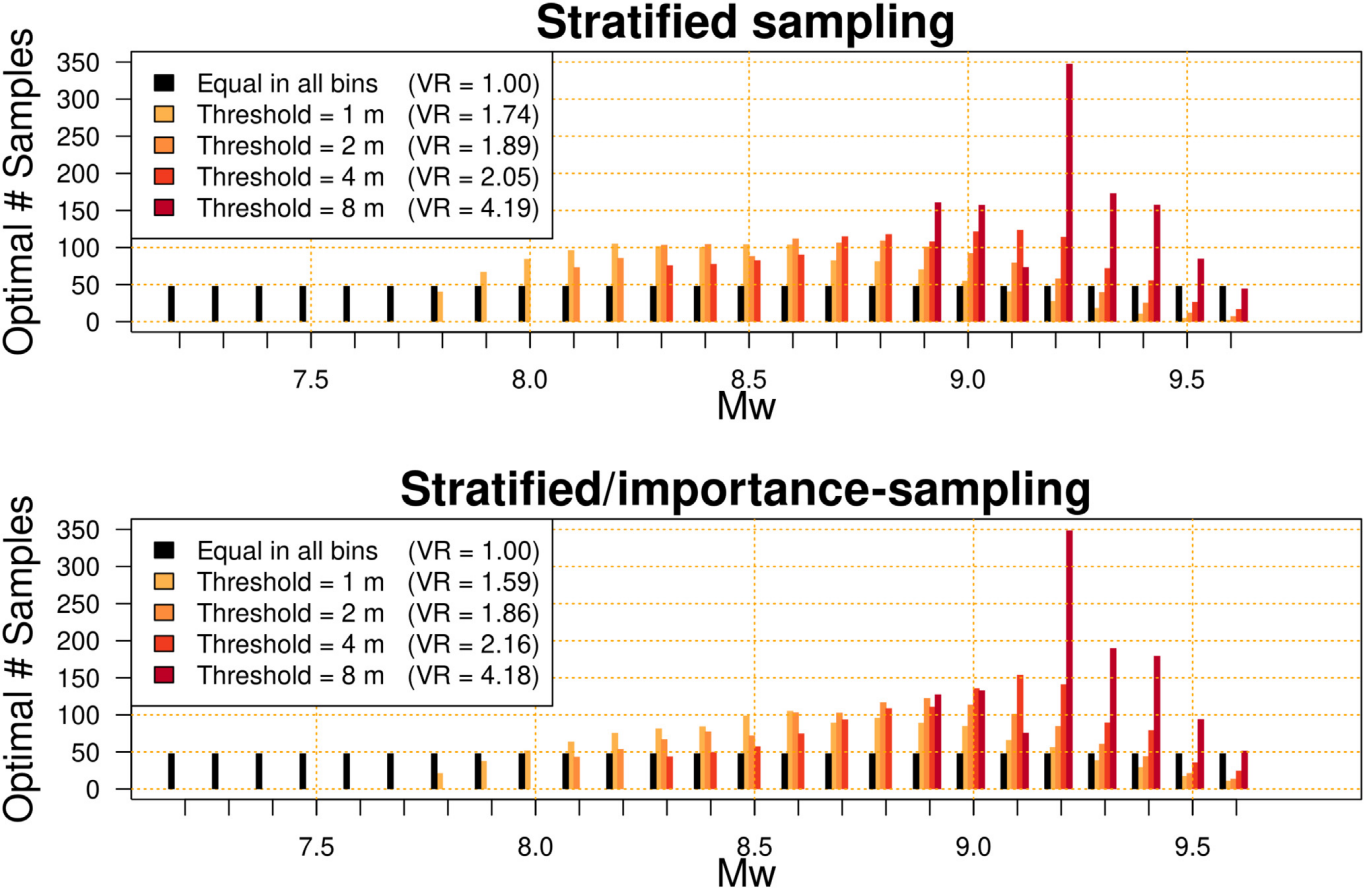
Optimal number of samples in each magnitude-bin for stratified-sampling (top panel) and stratified/importance-sampling (bottom panel), computed with eq. (31) for a range of thresholds *Q*^*T*^ at Site P. The logic-tree mean model $\overline{r}(e)$ was used to specify scenario-frequencies, and *N*_tot_ = 1200. VR gives the relative reduction in the Monte Carlo variance at each threshold due *only* to use of non-uniform *N*(*M*_w,*b*_). In the top panel VR compares stratified-sampling with uniform versus non-uniform *N*(*M*_w,*b*_). In the bottom panel VR compares stratified/importance-sampling with uniform versus non-uniform *N*(*M*_w,*b*_).

At Site P, by inspection it is clear that the optimal non-uniform sampling effort varies substantially with the selected threshold *Q*^*T*^ (Fig. 5). Both stratified and stratified/importance-sampling show a similar trend: at higher thresholds it is better to concentrate sampling on higher magnitudes. The efficiency improvement can be computed from the ratio of the variances when using uniform and non-uniform *N*(*M*_w,*b*_), while keeping other aspects of the sampling scheme fixed. This is quantified with VR in Fig. 5, where variances were computed with eq. (28). We stress these VR values show the *extra* reduction of variance due to non-uniform versus uniform *N*(*M*_w,*b*_), not the combined accuracy of each sampling technique. Stratified/importance-sampling will have much lower variance overall for reasons discussed above (Section 3.3.1), but here we only consider the improvement due to non-uniform *N*(*M*_w,*b*_). For the thresholds reported in Fig. 5, VR shows that non-uniform sampling reduces the variance by a factor of 1.6–4.2, for both stratified and stratified/importance sampling. There is greater potential for variance reduction at higher thresholds, because low-magnitude earthquakes are unlikely to generate large waves.

The full variance reductions in Fig. 5 cannot practically be obtained because only one set of *N*(*M*_w,*b*_) can be selected; the sampling effort cannot be tailored to every threshold and scenario-frequency model. Therefore, we suggest a compromise approach to determining non-uniform *N*(*M*_w,*b*_), informed by the optimal solutions and site-specific judgement:

- For robustness 1/4 of the scenarios are uniformly distributed among all magnitude-bins. This guarantees the Monte Carlo standard-deviation for *any* site or scenario-frequency model will never exceed twice the value that would have been obtained using uniform *N*(*M*_w,*b*_).
- The remaining 3/4 of scenarios are assigned non-uniformly, in proportion to the optimal *N*(*M*_w,*b*_) averaged over stage-thresholds of 1, 2, 4 and 8 m at the same Site P used to define the scenario importance (Fig. 5). These thresholds are expected to vary between applications. Herein they were chosen after some experimentation, because they cover mean exceedance-rates around 1/500–1/10 000 which are likely to be important in our application, and lead to reasonable variance reductions.

For these calculations we used the logic-tree mean scenario rate model $\overline{r}(e)$, restricted to thrust scenarios on the Kermadec-Tonga source. While other scenario-frequency models in *I* could be used, the mean model is a reasonable focus for optimization, as it is often used for inundation mapping at nominal return periods.

**Figure 6.**
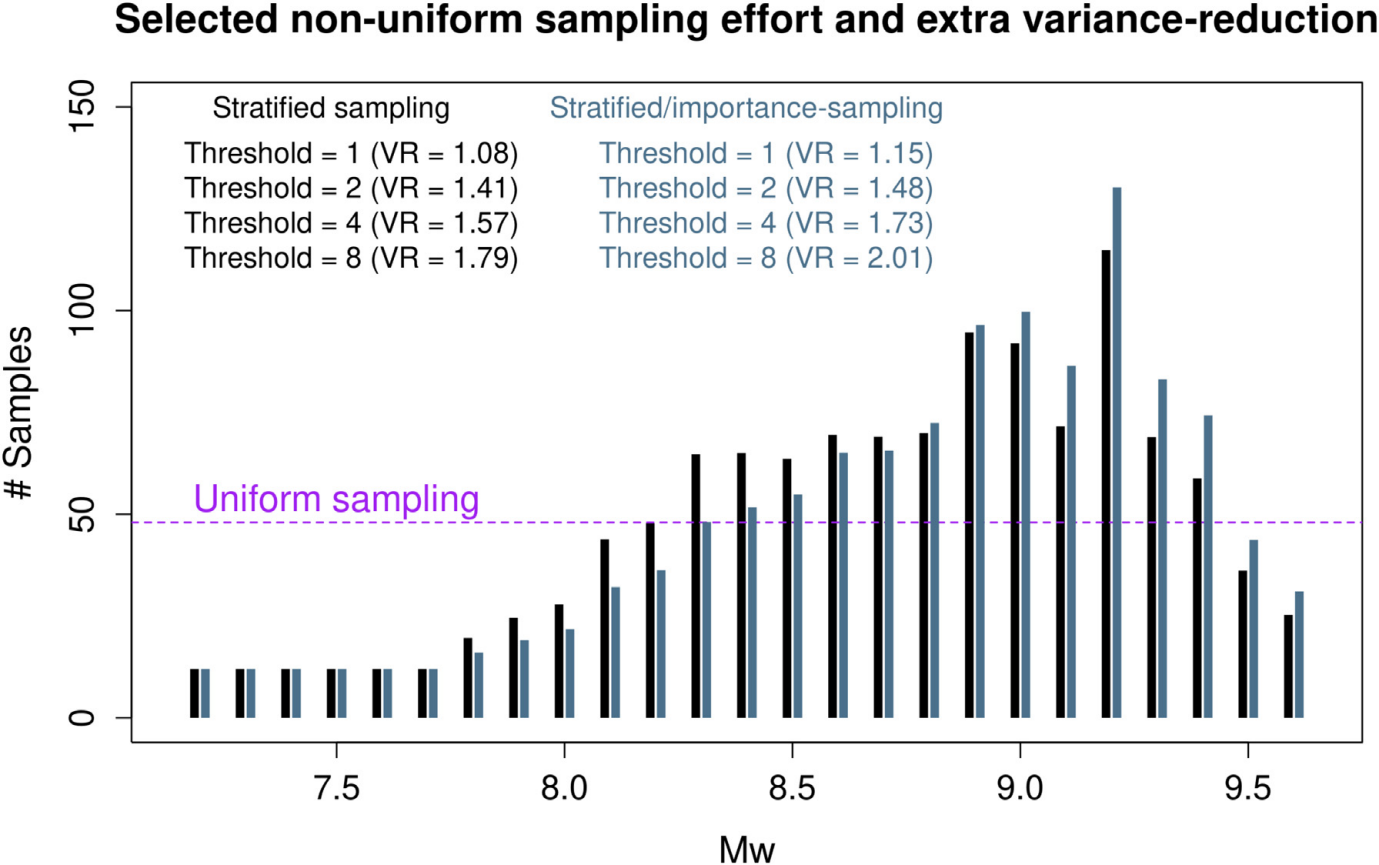
The selected number of samples in each magnitude-bin for both stratified-sampling and stratified/importance-sampling. This is a compromise between the optimal solutions for different *Q*^*T*^ values. VR gives the relative reduction in the Monte Carlo variance at the specified threshold due *only* to use of non-uniform *N*(*M*_w,*b*_). The black VR values compare stratified-sampling using uniform versus non-uniform *N*(*M*_w,*b*_), while the blue VR values compare stratified/importance-sampling using uniform versus non-uniform *N*(*M*_w,*b*_). The results apply to the offshore Site P that was used to define the scenario importance, with exceedance-rates evaluated using the logic-tree mean scenario-frequency model, and *N*_tot_ = 1200.

The resulting non-uniform *N*(*M*_w,*b*_) leads to more sampling at *M*_w_ 8.3–9.4, and less sampling at low and very high magnitudes (Fig. 6). The associated variance-reductions are necessarily less than the optimal VR for the same threshold (compare VR values in Figs 6 and 5). However they are still equivalent to using 1.1–2.0 times more scenarios, relative to using uniform *N*(*M*_w,*b*_) (Fig. 6). The benefit of non-uniform sampling increases at higher threshold values, which is desirable because larger tsunamis tend to have greater Monte Carlo errors (Figs 2 and 3). In practice, we should also check the performance at sites other than Site P; this is the focus of Section 3.5.

### 3.5 Testing the sampling scheme at offshore sites prior to inundation simulation

Before selecting a sampling scheme and running expensive inundation computations we recommend testing the approach at nearby offshore sites, using the offshore PTHA. This has minimal computational cost and allows any problems with the sampling scheme to be identified and fixed prior to expensive tsunami simulation. Here the new Monte Carlo scheme is compared with regular stratified-sampling with uniform *N*(*M*_w,*b*_), which represents the standard approach. In both cases *N*_tot_ = 1200. The new scheme combines stratified/importance-sampling based on Site P with non-uniform *N*(*M*_w,*b*_) (Fig. 6). The same Monte Carlo sample is reused for both segmented and unsegmented source models, as explained in Section 3.3.2. Section 4 uses the same sampling scheme for probabilistic inundation computations in Tongatapu.

**Figure 7.**
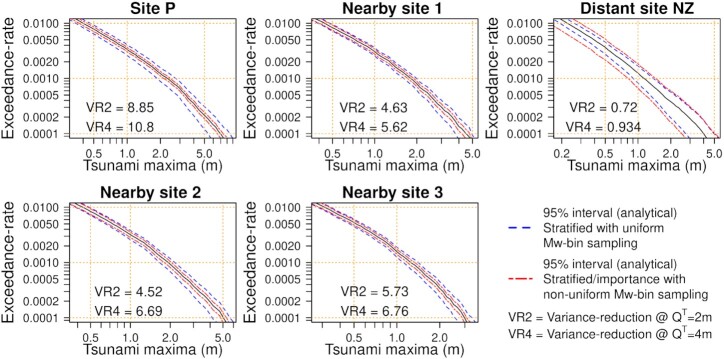
Monte Carlo variability in the logic-tree mean exceedance-rate curves at offshore sites. Regular stratified-sampling with uniform *N*(*M*_w,*b*_) is compared with the new scheme (stratified/importance-sampling with non-uniform *N*(*M*_w,*b*_), details matching Fig. 6). The 95 per cent intervals and relative variance reductions were derived analytically (eqs 12 and 23), assuming a normal distribution for the confidence intervals. Note the VR2 and VR4 values show the efficiency improvements of stratified/importance-sampling with non-uniform *N*(*M*_w,*b*_), relative to stratified-sampling with uniform *N*(*M_w, b_*).

Fig. 7 depicts 95 per cent confidence-intervals for the logic-tree mean hazard curves at the same sites used in Fig. 4. The relative variance reductions at *Q*^*T*^ = 2, 4 m compare the variance of the new scheme with the variance of the standard approach (Fig. 7). They show the new scheme improves the efficiency by a factor of 4.5–10.8 near Tongatapu, with all sites showing a greater efficiency improvement for larger waves. At distant site NZ there is a small efficiency reduction, reflecting that stratified/importance sampling can reduce the efficiency far from Site P (which was used to define the scenario importance $\mathcal {I}(e)$ and non-uniform *N*(*M*_w,*b*_) values). But near Tongatapu the improvements are substantial.

**Figure 8.**
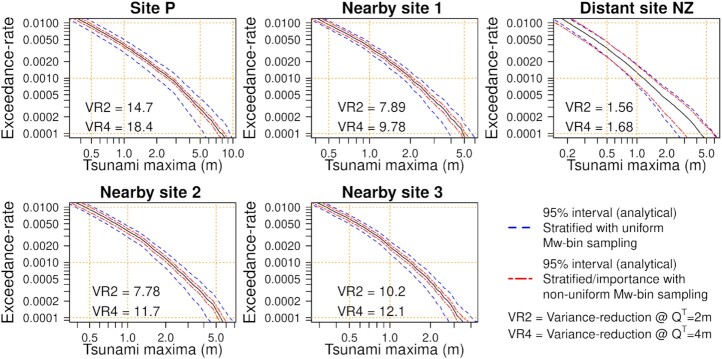
Monte Carlo variability in the exceedance-rate curves at offshore sites with 100 per cent weight on the unsegmented scenario-frequency model. Other details are the same as Fig. 7 except stratified-sampling uses 600 scenarios (assuming the other half would be used to sample the segmented models). Stratified/importance-sampling uses 1200 scenarios with the same sampling approach as Fig. 7.

To highlight the benefits of reusing scenarios for epistemic uncertainty calculations, the calculations were repeated with 100 per cent weight on the unsegmented source-zone representation (Fig. 8). The stratified/importance sampling strategy was unchanged, matching the approach used in practice (Section 4). Epistemic uncertainty calculations via stratified-sampling require that the 1200 scenarios are shared among the four source models (unsegmented and three segments; Section 3.2.2). Thus the stratified-sampling results in Fig. 8 assume only 600 of the 1200 Monte Carlo scenarios are used to sample the unsegmented model. This is a fair comparison because in practice, the other 600 Monte Carlo scenarios would be used to sample the segmented models, which are assigned 50 per cent weight in PTHA18.

Fig. 8 shows that stratified/importance-sampling leads to even greater efficiency improvements for the unsegmented model (Fig. 8), as compared to the improvements observed for the logic-tree-mean model (Fig. 7). Sites near Tongatapu exhibit variance reductions of a factor 7.7–18.4 at thresholds *Q*^*T*^ = 2, 4 m, with greater improvements for larger tsunamis. In this case even distant site NZ exhibits a small efficiency improvement. This highlights the benefit of Monte Carlo sample reuse when calculating epistemic uncertainties.

The results in Figs 7 and 8 confirm that the sampling scheme is likely to perform well near Tongatapu. This provides confidence in the applicability of our stratified/importance-sampling approach. The following section applies the technique to onshore hazard assessment.

## 4 APPLICATION TO PROBABILISTIC INUNDATION COMPUTATION

### 4.1 Tsunami scenarios and inundation computation

A single Monte Carlo sample with 1200 scenarios was constructed using stratified/importance-sampling with non-uniform *N*(*M*_w,*b*_) based on Site P, exactly matching the approach in Section 3.5. Although we only sampled thrust scenarios from the Kermadec-Tonga trench, a more comprehensive hazard assessment could include scenarios from other PTHA18 source-zones. This would increase the frequency of smaller tsunamis, and could be done by repeating the computations herein for multiple source-zones. Alternatively, multiple source-zones could be sampled at once by combining all in the set of scenarios *E*, using the same equations presented in Sections 3.3 and 3.4.

For all randomly sampled scenarios, the tsunami was simulated from source through to inundation with the shallow water equations using ocean-surface perturbations from PTHA18 (Davies & Griffin 2018). They were applied instantaneously to both the initial ocean surface, and the elevation. Scenarios were run with an initial (pre-earthquake) sea level of 0 m, and separately 0.8 m, to approximate the mean-sea level and monthly tidal maxima at Tongatapu. This gives some insight into the importance of tides; a more comprehensive treatment of tides is beyond the scope of this study (Lane *et al*. 2012; Adams *et al*. 2015; Wilson & Power 2020; González *et al*. 2021).

**Figure 9.**
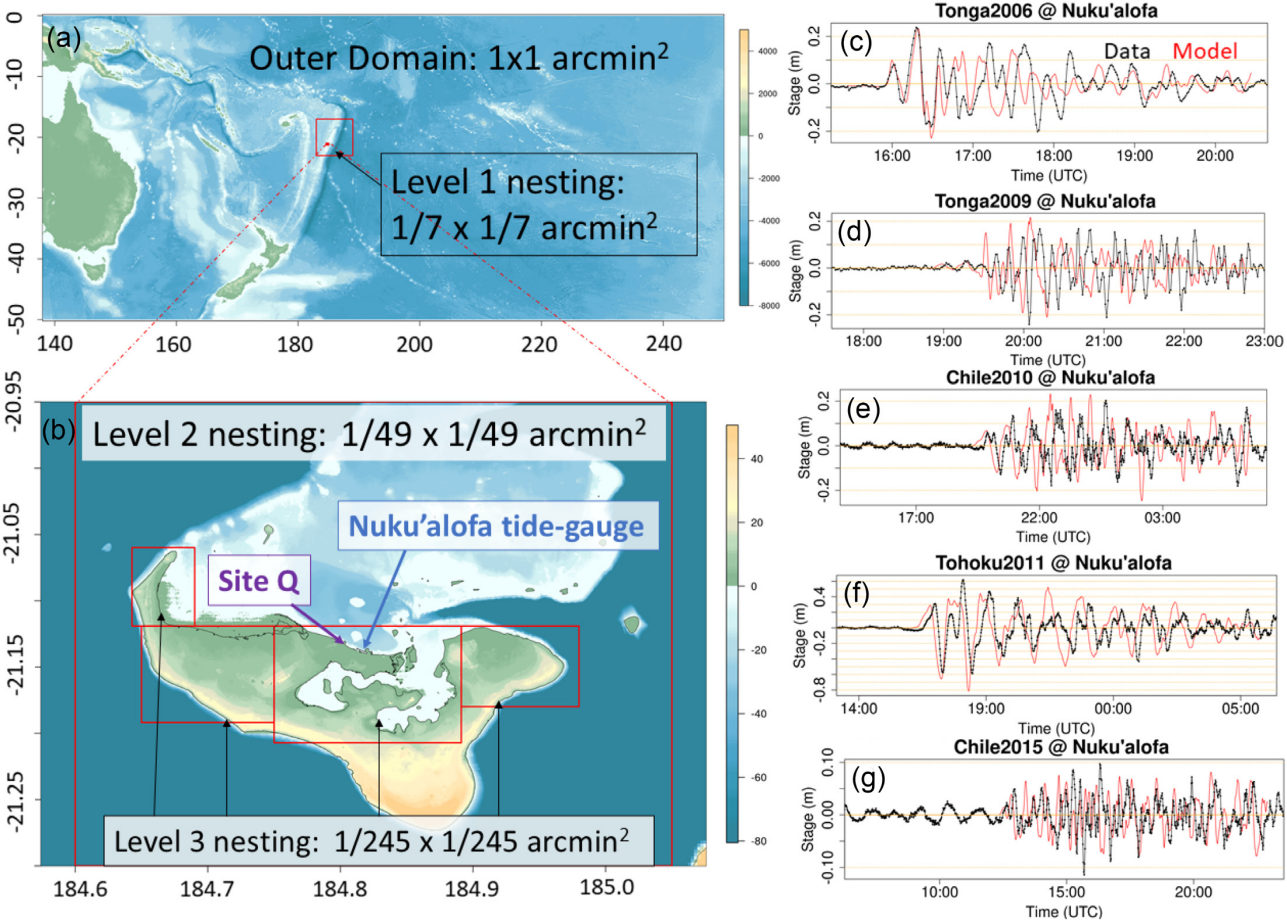
Model structure and comparison of models with historic tsunamis at Nuku’alofa tide-gauge. (a) Outer domain used to simulate all scenarios originating from the Kermadec-Tonga source. To simulate historic tsunamis from Chile and Tohoku, the outer domain was extended to include the tsunami source. (b) Model design near Tongatapu, with locations mentioned in the text. Black solid line is the OpenStreetMap coastline (© OpenStreetMap contributors). The highest resolution domains cover low-lying built-up areas of northern Tongatapu. (c–g) Comparison of historic tsunamis and models at Nuku‘alofa tide-gauge. All model results are derived from PTHA18 scenarios with earthquake magnitude and location similar to the historical event (Davies 2019).

The model domain (Fig. 9) uses three levels of two-way grid nesting to resolve inundation around Tongatapu with ≃7.5 m cell size. The shallow water solver SWALS was used (Davies *et al*. 2020) and detail on the numerical model setup is provided in the Supplementary Material, Section C. To check the model performance we simulated five historical tsunami events (Figs 9c–g), using initial conditions from PTHA18 scenarios that fit DART buoy observations relatively well (identified in Davies 2019). For each historical event the model gives a reasonable representation of the tsunami size and arrival time, even though the PTHA18 scenarios were selected from the randomly generated database and have not been tuned to reproduce observations at Tongatapu (Fig. 9). Improved agreement between the modelled and observed waveforms could likely be obtained via refinement of the source models (Bosserelle *et al*. 2020) and inclusion of loading and dispersion in the shallow water model (Allgeyer & Cummins 2014; Watada *et al*. 2014; Baba *et al*. 2017).

### 4.2 Compatibility with the offshore PTHA

**Figure 10.**
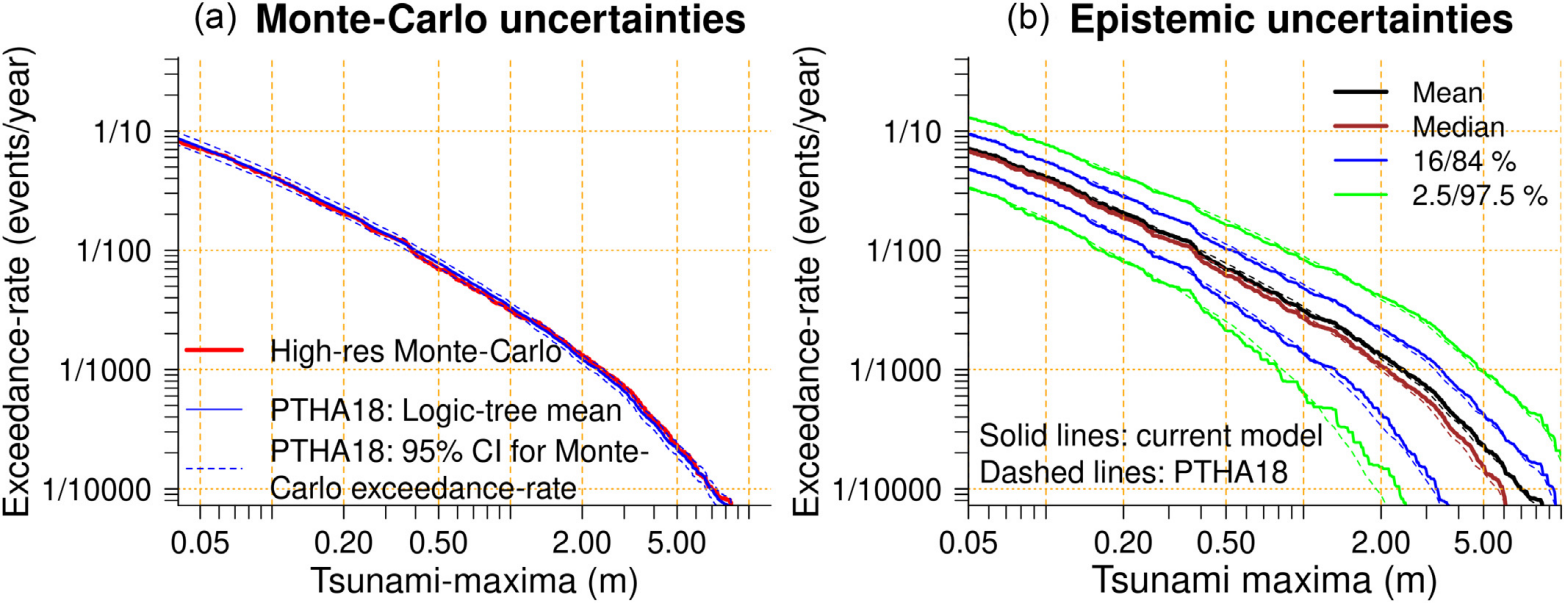
Consistency of the original PTHA18 and the Monte Carlo results at offshore Site P, using Kermadec-Tonga thrust scenarios and a background sea level of 0 m. (a) The maximum-stage exceedance-rate curve for the logic-tree mean model, as computed in PTHA18, and with the high-resolution Monte Carlo model. The 95 per cent confidence intervals were derived analytically from the PTHA18, assuming Monte Carlo errors for the sampling scheme are normally distributed with the mean and variance derived from eqs (1) and (23). (b) Comparison of epistemic uncertainties in the tsunami-maxima exceedance-rates from the PTHA18 (dashed lines) and the Monte Carlo results herein (solid lines).

To check the results we compared the offshore PTHA18 with the high-resolution Monte Carlo results at Site P (Fig. 10). This is an important test because, in principle, the exceedance-rate curves should agree well at deep water sites far from the coast, where the linear assumptions underling the offshore PTHA are reasonable (Gibbons *et al*. 2020; Tonini *et al*. 2021). Significant differences would warrant investigation because they could indicate implementation errors, or the use of too small a Monte Carlo sample with the high-resolution model. Conversely, they might simply reflect differences in the hydrodynamic models and elevation data used in both studies (Gibbons *et al*. 2020). For example, compared to our high-resolution model (Section 4.1) the PTHA18 hydrodynamic model is much simpler; it solves the frictionless linear shallow water equations at a globally uniform 1 arcmin resolution for 36 hr duration (Davies & Griffin 2020).

Despite differences in their hydrodynamics, Fig. 10 shows there is good agreement between the high-resolution Monte Carlo results and the PTHA18 results at Site P. For the logic-tree mean exceedance-rate curve, the high-resolution Monte Carlo results are largely within the 95 per cent analytical Monte Carlo confidence-interval derived from the offshore PTHA (Fig. 10a). There is also good agreement between percentiles of epistemic uncertainties as derived in both studies (Fig. 10b). These epistemic uncertainty calculations follow the methodology of PTHA18, which uses a conservative model of dependence between the Tonga, Kermadec and Hikurangi segments (details in Davies & Griffin 2020). The agreement in Fig. 10 gives confidence that the high-resolution Monte Carlo calculations have been correctly implemented.

Greater differences were reported in a similar test by Gibbons *et al*. (2020) using the TSUMAPS-NEAM offshore PTHA and a set of more than 30 000 high-resolution scenarios (selected with non-random sampling). This may reflect that their comparison site was located in relatively shallow water (50 m deep) about 6 km offshore. The effects of non-linear hydrodynamics and high-resolution bathymetry are likely to be more pronounced at such a site, as compared to Site P which is 18 km offshore Tongatapu in 800 m depth. Because nearshore and shallow-water effects make it harder to note problematic differences between the offshore and nearshore PTHAs (e.g. caused by implementation errors or insufficient sampling), we recommend testing at a deep-water site far from the coast, if available in the offshore PTHA.

### 4.3 Probabilistic inundation: Monte Carlo accuracy and epistemic uncertainty

**Figure 11.**
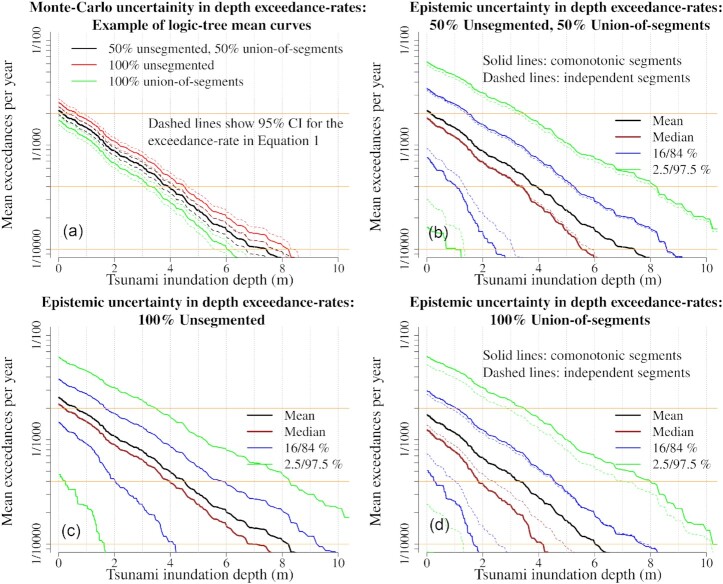
Monte Carlo and epistemic uncertainties in inundation depth exceedance-rates at Site Q (lon,lat = 184.8017,–21.13305). The background sea level is 0 m and only Kermadec-Tonga trench thrust scenarios are included. (a) Logic-tree-mean curves for 3 different scenario-frequency representations, with 95 per cent confidence-intervals (computed via eq. 25) for the exceedance-rate in eq. (1). (b) Epistemic uncertainties in the inundation-depth exceedance-rates. Percentiles vary slightly if the segments are treated as comonotonic (solid lines) or independent (dashed lines). (c) Same as panel (b) but with 100 per cent weight on the unsegmented source representation. (d) Same as panel (b) but with 100 per cent weight on the union-of-segments source representation.

To study the Monte Carlo accuracy onshore we consider ‘Site Q’ in Tongatapu, which is inland about 150 m from the shoreline (location in Fig. 9b). Similar analyses can be applied to any onshore site of interest. Depth exceedance-rates were computed for the logic-tree mean model, and 95 per cent confidence-intervals for the exceedance-rate in eq. (1) were computed with eq. (25) (Fig. 11a). Results with 100 per cent weight on the unsegmented or union-of-segments source representations are also shown (Fig. 11a). The 95 per cent exceedance-rate intervals range over ≃9 per cent of the estimated exceedance-rate at 1/500, growing slowly to ≃11 per cent at 1/2500 and ≃16 per cent at 1/10 000. These numbers are similar for all scenario-frequency models in Fig. 11(a). If the high-resolution Monte Carlo calculations were repeated many times with different random scenarios, then the exceedance-rate (eq. 1) for each source-model would be within these intervals approximately 95 per cent of the time.

For a fixed exceedance-rate, Monte Carlo uncertainties in the depth can also be inferred from horizontal transects along Fig. 11(a). For the logic-tree mean model, the upper limit of the 95 per cent interval differs from the computed mean by +16 cm (at 1/500 exceedance-rate), +22 cm (1/2500), and +33 cm (1/10 000). Based on the offshore results (Section 3.5) we anticipate these intervals would be ≃2−4.3 times wider if the calculations had used regular stratified-sampling with uniform *N*(*M*_w,*b*_) and the same computational effort; in that case it would be difficult to distinguish the different source representations in Fig. 11(a).

The Monte Carlo confidence-intervals in Fig. 11(a) reflect the expected error given a limited number of random scenarios (*N*_tot_ = 1200) and could be reduced using a larger Monte Carlo sample. However, they are already small compared to the epistemic uncertainties (Fig. 11b) which reflect our uncertain knowledge of earthquake magnitude-frequency relations on the Kermadec-Tonga trench (Figs 1c–f). In tsunami risk management practice the 84th percentile depth is sometimes used in preference to the mean (Leonard *et al*. 2008; MCDEM 2016; Tonini *et al*. 2021); in Fig. 11(b) the 84th percentile curve exceeds the mean curve by a depth of 1−2 m for a given exceedance-rate. That difference is substantially greater than the Monte Carlo uncertainties (Fig. 11a) suggesting limited practical benefit to using a larger *N*_tot_ at this site, given our novel sampling scheme.

Some conceptual subtleties arise in the epistemic uncertainty calculations (Figs 11b–d), depending on how the segmented models are combined. To calculate epistemic uncertainties the Monte Carlo exceedance-rate estimates (eq. 22) are first computed for all logic-tree scenario-frequency models (corresponding to individual grey curves in Figs 1c–f). Each scenario-frequency model has a Bayesian posterior weight determined as in Davies & Griffin (2020), and for any depth threshold these combine to give a distribution of exceedance-rates. There is one such distribution for the unsegmented model, and one for each of the three segments. Percentiles can be computed to summarize these distributions for any depth threshold, as depicted for the unsegmented model in Fig. 11(c). However, the union-of-segments case (Fig. 11d) is more complex because three segments lead to three distributions which must be combined, and the distribution of their union is affected by any dependence between the segments (although the mean is unaffected). Davies & Griffin (2020) argued that dependence in epistemic-uncertainties should be used to reflect the possibility that similar physical processes control maximum earthquake magnitudes, coupling, etc. This increases the likelihood that relatively high (or low) epistemic-uncertainty percentiles are true on all segments simultaneously. PTHA18 conservatively accounts for this by assuming comonotonic dependence between segments, that is if the 84th percentile exceedance-rate is true on one segment, it is true on all segments simultaneously (and similarly for other percentiles, Deelstra *et al*. 2009). While the comonotonic approach is also used herein, to better understand its effect we repeated the calculations assuming independent segments (Fig. 11d). The intersegment dependence treatment also affects the full model (Fig. 11b) because it is a 50:50 sum of the unsegmented and union-of-segments probability densities. However at this site the effects of comonotonic dependence versus independence of segments are small for higher percentiles, although significant for lower percentiles (Figs 11b and d). This is because higher percentiles are predominantly affected by one segment (Tonga) which is near-field; the other two segments are less likely to generate large waves at Tongatapu, so contribute much less to high-hazard percentiles irrespective of intersegment dependence. While the dependence treatment does not have a major influence on epistemic uncertainties at this site, the impacts could be greater elsewhere if multiple segments contributed more equally to the hazard.

**Figure 12.**
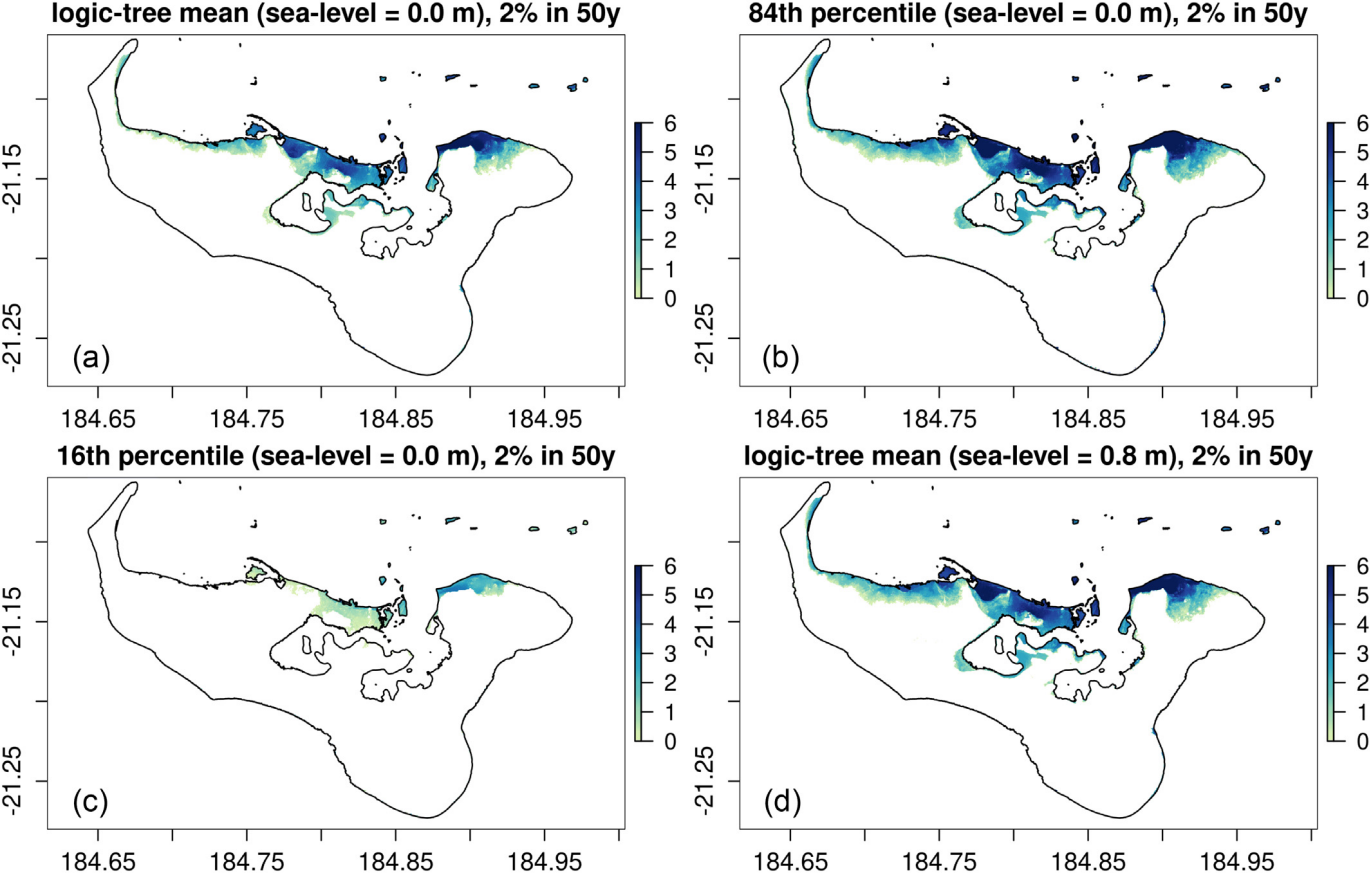
Inundation depth with a 2 per cent chance of exceedance in 50 yr. Solid line is the OpenStreetMap coastline (© OpenStreetMap contributors). (a) Logic-tree mean with background sea level of 0 m. (b) 84th percentile of the epistemic uncertainty with a background sea level of 0 m. (c) 16th percentile of the epistemic uncertainty with a background sea level of 0 m. (d) Logic-tree mean with a background sea level of 0.8 m.

The spatial distribution of depths with exceedance-rate of 1/2475 (equivalent to a 2 per cent chance of exceedance in 50 yr) were computed for the logic-tree mean curve as well as the 84th and 16th percentiles (Figs 12a–c). This was implemented by repeating site-specific calculations like in Fig. 11 at each pixel, using root-finding (Brent 1973) to find *Q*^*T*^ values corresponding to the desired exceedance-rate. The difference between the 16th and 84th percentile results (Figs 12b and c) highlight the large epistemic uncertainties in inundation depths, which stem from large uncertainties in PTHA18's magnitude–frequency relations for the Kermadec-Tonga trench (Figs 1c–f). Low-lying areas along the north coast are inundated in all cases, while elsewhere, the steep coastal topography of Tongatapu limits the inundation extent.

The results in Figs 12(a)–(c) assume a background sea level equal to mean-sea level, whereas in practice tides at Tongatapu have a typical monthly maxima of 0.8 m. If the tsunami maxima were to coincide with such a high-tide then the logic-tree mean depths increase (compare Figs 12d and a) but are still smaller than the 84th percentile depths with a background sea level of 0 m (Fig. 12b). Although not undertaken herein, the results suggest that while tidal aleatory uncertainties are significant at Tongatapu, they are less influential for understanding tsunami hazards than epistemic uncertainties in source frequencies. However by 2100 sea levels at Nuku’alofa are projected to rise by approximately 0.8 m due to climate change (Fox-Kemper *et al*. 2021), so in future we expect an increasing chance of relatively high sea levels exacerbating tsunami inundation (Li *et al*. 2018; Sepúlveda *et al*. 2021).

## 5 CONCLUSIONS

Offshore PTHAs are increasingly available globally and provide a foundation for high-resolution onshore PTHAs, which are useful to guide tsunami risk-management. To translate the offshore hazard to an onshore site of interest, ideally high-resolution tsunami simulations would be conducted for every scenario in the offshore PTHA, but this is usually computationally prohibitive. Monte Carlo sampling offers an attractive alternative that converges to the latter solution, and can have acceptable accuracy at much lower computational cost. Theoretical results enable the performance of all sampling schemes considered herein to be understood at offshore sites (where the offshore PTHA is valid) prior to sampling. This enables proposed Monte Carlo sampling schemes to be checked, and perhaps further optimized, prior to expensive tsunami computation. Furthermore, once the inundation computations are available, the Monte Carlo error at coastal sites can be estimated from the single Monte Carlo sample, and offshore results can be cross-checked with the offshore PTHA. In combination these techniques facilitate the control of Monte Carlo errors when translating from offshore to onshore PTHA.

The key novel contributions of this paper involve using the offshore PTHA to improve the Monte Carlo efficiency near a site of interest. Stratified/importance-sampling enables better sampling of scenarios that are likely to be important according to user-defined criteria (based on offshore tsunami maxima herein). Compared to stratified-sampling this can substantially reduce Monte Carlo errors for a given computational cost, both because it better represents important scenarios, and because it facilitates greater reuse of the Monte Carlo sample in epistemic uncertainty calculations. Further efficiency improvements were obtained via non-uniform sampling of magnitude-bins, informed by theoretically optimal solutions near the site of interest. The latter technique can be combined with both stratified and stratified/importance-sampling. In our application, the combination of these techniques led to efficiency improvements near Tongatapu, equivalent to using ≃4−18 times more scenarios with uniform stratified-sampling. These efficiency improvements vary from site to site but are consistently greater for rare return periods, and quantification of epistemic-uncertainties. Because onshore PTHA is often limited by the computational cost, this will help enable PTHA to be used for a greater range of onshore hazard applications.

In future it would be interesting to try combining the techniques here with fast approximate inundation computation (e.g. de Baar & Roberts 2017; Guillas *et al*. 2018; Volpe *et al*. 2019; Williamson *et al*. 2020; Fukutani *et al*. 2021; Makinoshima *et al*. 2021). This will involve some trade-offs that remain to be fully explored; for example, while Monte Carlo errors could be reduced using many approximate inundation scenarios, the latter may introduce systematic errors that offset the benefit. In such cases the approximate inundation may still be of value to guide the scenario importance definition. There is also scope to extend stratified/importance-sampling to more complex situations. For example, dynamic tides might be treated by considering the tidal-phase as a random variable and integrating it into the user-defined scenario importance, to reflect that greater inundation is expected near high-tides. The techniques could also be extended to other kinds of tsunami sources (e.g. landslides, volcanoes), if useful definitions of the scenario importance can be invented. Further consideration should also be given to the treatment of multiple source-zones, as our method offers two approaches to this. One is to combine multiple sources into the set of scenarios *E* prior to sampling; the other is to sample each source-zone separately with a chosen number of scenarios. The former approach will cause the number of scenarios on each source to be randomly determined, with better representation of source zones that often produce important scenarios, but potentially limited representation of others. The latter approach gives the modeller control over the representation of each source zone, and would also permit the use of varying scenario importance definitions for each.

Finally, we stress key limitations to this work. Although our techniques are general, the probabilistic inundation hazard results presented here only reflect PTHA18 thrust scenarios on the Kermadec-Tonga source zone, and neglect outer-rise earthquakes, more distant earthquake sources, landslides and volcanoes. Both palaeotsunami results and the recent 15/01/22 Hunga-Tonga volcanic tsunami highlight the significance of such sources for hazards in Tongatapu (Frohlich *et al*. 2009; Lavigne *et al*. 2021; Duncombe 2022). The model does not account for dynamic tides (Wilson & Power 2020), future mean-sea level changes (Sepúlveda *et al*. 2021), or other sources of prediction errors in non-linear shallow water models such as imperfectly known bathymetry and simplification of the three-dimensional physics (Bosserelle *et al*. 2020; Sepúlveda *et al*. 2020; Tonini *et al*. 2021). While sufficient to demonstrate the performance of our offshore-to-onshore Monte Carlo techniques, the probabilistic inundation results imply large uncertainties in the thrust sources. Reducing these uncertainties would require uncertainty reductions in the offshore PTHA. One way to approach this is via better integration of onshore tsunami observations into the offshore PTHA (e.g. older historical and palaeotsunami data). To this end it may be useful to leverage efficient techniques for translating the offshore PTHA onshore, such as proposed herein, to enable more rigorous comparison of probabilistic models and onshore data.

## Supplementary Material

ggac140_Supplemental_File

## Data Availability

The code and tsunami observations used for this study are available at https://github.com/GeoscienceAustralia/ptha/tree/master/misc/monte_carlo_paper_2021.
