# Supplementary material for: From offshore to onshore probabilistic tsunami hazard assessment via efficient Monte Carlo sampling
Source: Geophys J Int. 2022 Apr 11;230(3):1630–51. doi: 10.1093/gji/ggac140 (PMC9071009; doi:10.1093/gji/ggac140)
Supplement: ggac140_Supplemental_File [file ggac140_supplemental_file.pdf]

Supplementary Material for 'From offshore to onshore PTHA via efficient Monte-Carlo sampling'

G. Davies, R. Weber, K. Wilson, P. Cummins

A Stratified-sampling: Further explanation of Equations 9 and 10

For stratified-sampling, Equations 9 and 10 describe the variance of the within-magnitude-bin Monte-Carlo exceedance rate (Equation 7).

- Equation 9 gives the exact variance of Equation 7
- Equation 10 gives an estimate Equation 9, which can be computed using only the sampled scenarios.

Below we provide a proof that Equation 9 is the variance of Equation 7, and further explain the ideas used to approximate Equation 9 with Equation 10.

A.1 Proof that Equation 9 is the variance of Equation 7

As noted in the Section 3.2, Equation 7 can be written as:

$$\widehat{\lambda_i^{SS}}(Q > Q^T|M_{w,b}) = \Big[\frac{\lambda_i(M_{w,b})}{N(M_{w,b})}\Big]\mathcal{B}\big(p_{b,i,T}, N(M_{w,b})\big) \tag{A.1}$$

where $\mathcal{B}\big(p_{b,i,T}, N(M_{w,b})\big)$ is a binomial random variable with probability $p_{b,i,T}$ and size $N(M_{w,b})$. The binomial term has mean $\Big(p_{b,i,T}N(M_{w,b})\Big)$ and variance $\Big(N(M_{w,b})p_{b,i,T}(1-p_{b,i,T})\Big)$ (e.g., Bolker, 2008).

We wish to prove that Equation 9 is the variance of Equation A.1. Equation A.1 is a constant multiple of a binomial random variable. For any random variable X and constant k, if the variance of X is $\sigma^2(X)$ then the variance of kX is $k^2\sigma^2(X)$. This follows directly from the definition of the variance (e.g. Bolker, 2008).

Applying these results to calculate the variance of Equation A.1 gives:

$$\sigma^2\Big(\widehat{\lambda_i^{SS}}(Q > Q^T|M_{w,b})\Big) = \Big[\frac{\lambda_i(M_{w,b})}{N(M_{w,b})}\Big]^2\sigma^2\Big(\mathcal{B}\big(p_{b,i,T}, N(M_{w,b})\big)\Big) \tag{A.2}$$

$$= \Big[\frac{\lambda_i(M_{w,b})}{N(M_{w,b})}\Big]^2 N(M_{w,b})p_{b,i,T}(1-p_{b,i,T}) \tag{A.3}$$

$$= \frac{\Big(\lambda_i(M_{w,b})\Big)^2}{N(M_{w,b})}p_{b,i,T}(1-p_{b,i,T}) \tag{A.4}$$

which gives Equation 9.

A.2 Justification of Equation 10

Equation 10 is an estimate of Equation 9 that can be computed using only the sampled scenarios. To enable this we simply replace $p_{b,i,T}$ in Equation A.4 with an estimate $\widehat{p}_{b,i,T}$ that is derived from the fraction of exceedances in the sampled scenarios.

B Stratified/importance-sampling: Further explanation of Equations 19 and 20

For stratified/importance-sampling, Equations 19 and 20 describe the variance of the within-magnitude-bin Monte-Carlo exceedance rate (Equation 17).

This section provides a proof that the variance of Equation 17 is equal to Equation 19. We also provide further explanation of the approximations used to obtain Equation 20 as an estimate of Equation 19.

B.1 Preliminary results

The following standard results are used in the subsequent arguments.

For a discrete random variable X with probability mass function $\rho(x)$, the mean of X is:

$$\mu(X) = \sum_{x \in X} \rho(x) x \tag{A.5}$$

and the variance of X is:

$$\sigma^2(X) = \sum_{x \in X} \rho(x)(x - \mu)^2 \tag{A.6}$$

(e.g. Bolker, 2008).

Basic importance-sampling (e.g. Owen & Zhou, 2000; Tokdar & Kass, 2009; Lie & Quer, 2017) can be used to approximate Equation A.5 using a weighted random sample drawn from X with replacement, where the sampling weights correspond to a chosen probability mass function $w^\tau(x)$, which can differ from $\rho(x)$. Denote this sample as X^τ, and assume it has sample size N. The basic importance-sampling estimate of $\mu(X)$ is:

$$\mu(X) \simeq \frac{1}{N} \sum_{x \in X^\tau} \frac{\rho(x)}{w^\tau(x)} x \tag{A.7}$$

In our context, the offshore PTHA gives the within-magnitude-bin exceedance-rate as:

$$\lambda_i(Q > Q^T | M_{w,b}) \quad = \quad \lambda_i(M_{w,b}) \sum_{e \in E_b} w_{b,i}^{SS}(e) \mathbb{1}_{(Q(e) > Q^T)} \tag{A.8}$$

where $w_{b,i}^{SS}(e)$ gives the within-magnitude-bin conditional probability of scenario e (note for stratified-sampling, $w_{b,i}^{SS}$ is the same as the sampling weights, see Equation 6). If the scenarios are instead sampled with alternative weights $w_{b,i}^{SIS}(e)$, then from Equation A.7 the basic importance-sampling estimate of Equation A.8 is:

$$\widehat{\lambda_i}(Q > Q^T | M_{w,b}) = \frac{\lambda_i(M_{w,b})}{N(M_{w,b})} \sum_{e \in E_{b,i}^{SIS}} \frac{w_{b,i}^{SS}(e)}{w_{b,i}^{SIS}(e)} \mathbb{1}_{(Q(e) > Q^T)} \tag{A.9}$$

which is the same as Equation 17:

$$\widehat{\lambda_i}(Q > Q^T | M_{w,b}) = \frac{\lambda_i(M_{w,b})}{N(M_{w,b})} \sum_{e \in E_{b,i}^{SIS}} \phi_{b,i}^{SIS}(e) \mathbb{1}_{(Q(e) > Q^T)} \tag{A.10}$$

B.2 Proof that Equation 19 is the variance of Equation 17

Below we directly compute the variance of Equation A.10 (which is the same as Equation 17) to show it produces Equation 19.

The following intermediate result is useful. Suppose we sample a *single scenario* from E_b with probability mass function $w_{b,i}^{SIS}(e)$. Consider the term inside the summation on the RHS of Equation A.10.

The mean of this term can be derived from Equation A.5:

$$\mu\Big(\phi_{b,i}^{SIS}(e)\mathbb{1}_{(Q(e)>Q^T)}\Big) = \sum_{e\in E_b} w_{b,i}^{SIS}(e)\phi_{b,i}^{SIS}(e)\mathbb{1}_{(Q(e)>Q^T)} \quad \text{(A.11)}$$

$$= \sum_{e\in E_b} \frac{r(e)\mathbb{1}_{(Q(e)>Q^T)}}{\sum_{e\in E_b} r(e)} \quad \text{(A.12)}$$

$$= p_{b,i,T} \quad \text{(A.13)}$$

and its variance is (using Equation A.6):

$$\sigma^2\Big(\phi_{b,i}^{SIS}(e)\mathbb{1}_{(Q(e)>Q^T)}\Big) = \sum_{e\in E_b} w_{b,i}^{SIS}(e)[\phi_{b,i}^{SIS}(e)\mathbb{1}_{(Q(e)>Q^T)} - p_{b,i,T}]^2 \quad \text{(A.14)}$$

Next suppose we sample $N(M_{w,b})$ scenarios. Consider the summation term on the RHS of Equation A.10. The variance of this sum is equal to the sum of the variances of the individual terms (because each sample is independent, Bolker, 2008):

$$\sigma^2\Big(\sum_{e\in E_{b,i}^{SIS}} \phi_{b,i}^{SIS}(e)\mathbb{1}_{(Q(e)>Q^T)}\Big) = \sum_{e\in E_{b,i}^{SIS}} \sigma^2\Big(\phi_{b,i}^{SIS}(e)\mathbb{1}_{(Q(e)>Q^T)}\Big) \quad \text{(A.15)}$$

$$= N(M_{w,b})\sigma^2\Big(\phi_{b,i}^{SIS}(e)\mathbb{1}_{(Q(e)>Q^T)}\Big) \quad \text{(A.16)}$$

$$= N(M_{w,b})\sum_{e\in E_b} w_{b,i}^{SIS}(e)[\phi_{b,i}^{SIS}(e)\mathbb{1}_{(Q(e)>Q^T)} - p_{b,i,T}]^2 \quad \text{(A.17)}$$

Finally, using the fact that for any random variable X and constant k it is true that $\sigma^2(kX) = k^2\sigma^2(X)$, the variance of Equation A.10 is:

$$\sigma^2\Big(\widehat{\lambda}_i(Q > Q^T|M_{w,b})\Big) = \sigma^2\Big(\frac{\lambda_i(M_{w,b})}{N(M_{w,b})}\sum_{e\in E_{b,i}^{SIS}} \phi_{b,i}^{SIS}(e)\mathbb{1}_{(Q(e)>Q^T)}\Big) \quad \text{(A.18)}$$

$$= \Big(\frac{\lambda_i(M_{w,b})}{N(M_{w,b})}\Big)^2\sigma^2\Big(\sum_{e\in E_{b,i}^{SIS}} \phi_{b,i}^{SIS}(e)\mathbb{1}_{(Q(e)>Q^T)}\Big) \quad \text{(A.19)}$$

$$= \Big(\frac{\lambda_i(M_{w,b})}{N(M_{w,b})}\Big)^2 N(M_{w,b})\sum_{e\in E_b} w_{b,i}^{SIS}(e)[\phi_{b,i}^{SIS}(e)\mathbb{1}_{(Q(e)>Q^T)} - p_{b,i,T}]^2 \quad \text{(A.20)}$$

$$= \frac{\lambda_i(M_{w,b})^2}{N(M_{w,b})}\sum_{e\in E_b} w_{b,i}^{SIS}(e)[\phi_{b,i}^{SIS}(e)\mathbb{1}_{(Q(e)>Q^T)} - p_{b,i,T}]^2 \quad \text{(A.21)}$$

The last step gives Equation 19.

B.3 Justification of Equation 20

Equation 20 is an estimate of Equation 19 derived from basic importance-sampling. The summation term on the RHS of Equation A.21 can be approximated using the basic importance-sampling estimator (Equation A.7) as follows:

- $\rho(x)$ in Equations A.5 and A.7 is replaced with $w_{b,i}^{SIS}(e)$
- x in Equations A.5 and A.7 would ideally correspond to $[\phi_{b,i}^{SIS}(e)\mathbb{1}_{(Q(e)>Q^T)} - p_{b,i,T}]^2$. But it involves the term $p_{b,i,T}$ which cannot be computed using only the sampled scenarios.
  - To work around this issue, in place of $p_{b,i,T}$ we use $\widehat{q}_{b,i,T}$, which estimates the former using only the sampled scenarios. So x in Equation A.7 is replaced with $[\phi_{b,i}^{SIS}(e)\mathbb{1}_{(Q(e)>Q^T)} - \widehat{q}_{b,i,T}]^2$.
- $w^\tau(x)$ in Equation A.7 is replaced with $w_{b,i}^{SIS}(e)$, and X^τ is replaced with $E_{b,i}^{SIS}$.

These substitutions lead directly to Equation 20.

C Further information on the inundation model setup

The model domain (Figure 9) uses three levels of two-way grid nesting. The outer domain has $\simeq$ 1850 m cell-size, while high resolution areas around Tongatapu have $\simeq$ 7.5 m cell size. Elevation data was derived from a mixture of lidar, gridded bathymetric surveys near Tongatapu (Damlamian et al., 2013), and global-scale data derived from GA250 (Whiteway, 2009) and GEBCO-2014 Grid (version 20150318).

Tsunamis were simulated using the open-source SWALS code, which provides a number of explicit shallow water solvers including the linear solver used for PTHA18 (Davies & Griffin, 2018, 2020). This code has a test-suite of more than 20 analytical, laboratory or field problems, including well known tsunami problems from NTHMP (2012) (excluding landslides) and other recent NTHMP problems (Park et al., 2013; Lynett et al., 2017; Macías et al., 2020; Gao et al., 2020). The model setup herein mirrors that used by Davies et al. (2020) to compare modelled and observed tsunamis at 16 Australian tide-gauges. On all nested grids a second-order finite-volume scheme is used to solve the full nonlinear shallow water equations with uniform Manning-friction of 0.03 (details similar to Davies & Roberts, 2015). On the outer domain the linear shallow water equations are combined with Manning-friction and solved using a classical scheme (Goto & Ogawa, 1997), with details matching Davies et al. (2020).

To check the model performance, a number of convergence tests were conducted by factor-of-two coarsening of the model resolution. This had a small impact on the hydrodynamic results, suggesting the model resolution is fine enough. We also simulated five historic earthquake-tsunamis measured on the Nuku'alofa tide-gauge (Figure 9). These resulted from earthquakes in Tonga 2006/05/03 (M_w8.0), Tonga 2009/09/29 (M_w8.1), Chile 2010/02/27 (M_w8.8), Tohoku 2011/03/11 (M_w9.1), and Chile 2015/09/16 (M_w8.3). For each historic event a similar tsunami scenario was selected from among PTHA18 scenarios that fit DART buoy observations relatively well (as identified in Davies, 2019). To simulate the Tohoku and Chile events the outer domain was extended to cover the Pacific Ocean and it was evolved for 24 hours. For the Tonga events the model was evolved for 5 hours with an outer-domain matching Figure 9, identical to that used to simulate the random scenarios.

References

Bolker, B. M., 2008. *Ecological Models and Data in R*, Princeton University Press, Princeton, NJ.

Damlamian, H., Cummins, P., Tokavou, N., Buikoto, L., 'Aho1, L., Sagar, S., Raj, A., & Powers-Tora, M., 2013. Tsunami Inundation Modelling Of Tongatapu, Kingdom of Tonga, URL: https://www.preventionweb.net/files/45270_219.pdf.

Davies, G., 2019. Tsunami variability from uncalibrated stochastic earthquake models: tests against deep ocean observations 2006-2016, *Geophysical Journal International*, **218**(3), 1939–1960.

Davies, G. & Griffin, J., 2018. The 2018 Australian Probabilistic Tsunami Hazard Assessment: Hazards from earthquake generated tsunamis., Tech. rep., Geoscience Australia Record 2018/41.

Davies, G. & Griffin, J., 2020. Sensitivity of Probabilistic Tsunami Hazard Assessment to Far-Field Earthquake Slip Complexity and Rigidity Depth-Dependence: Case Study of Australia, *Pure and Applied Geophysics*, **177**, 1521–1548.

Davies, G. & Roberts, S., 2015. Open source flood simulation with a 2D discontinuous-elevation hydrodynamic model, in *Proceedings of MODSIM 2015, Gold Coast*.

Davies, G., Romano, F., & Lorito, S., 2020. Global Dissipation Models for Simulating Tsunamis at Far-Field Coasts up to 60 hours Post-Earthquake: Multi-Site Tests in Australia, *Frontiers in Earth Science*, **8**, 497.

Gao, S., Collecutt, G., Syme, W. J., & Ryan, P., 2020. High resolution numerical modelling of tsunami inundation using quadtree method and GPU acceleration, in *Proceedings of the 22nd IAHR-APD Congress 2020, Sapporo, Japan*.

Goto, C. & Ogawa, Y., 1997. Numerical method of tsunami simulation with the leap-frog scheme, Tech. rep., IUGG/IOC Time Project.

Lie, H. C. & Quer, J., 2017. Some connections between importance sampling and enhanced sampling methods in molecular dynamics, *The Journal of Chemical Physics*, **147**(19), 194107.

Lynett, P. J., Gately, K., Wilson, R., Montoya, L., Arcas, D., Aytore, B., Bai, Y., Bricker, J. D., Castro, M. J., Cheung, K. F., David, C. G., Dogan, G. G., Escalante, C., González-Vida, J. M., Grilli, S. T., Heitmann, T. W., Horrillo, J., Kânoğlu, U., Kian, R., Kirby, J. T., Li, W., Macías, J., Nicolsky, D. J., Ortega, S., Pampell-Manis, A., Park, Y. S., Roeber, V., Sharghivand, N., Shelby, M., Shi, F., Tehranirad, B., Tolkova, E., Thio, H. K., Velioğlu, D., Yalçıner, A. C., Yamazaki, Y., Zaytsev, A., & Zhang, Y., 2017. Inter-model analysis of tsunami-induced coastal currents, *Ocean Modelling*, **114**, 14–32.

Macías, J., Castro, M. J., Ortega, S., & González-Vida, J. M., 2020. Performance assessment of Tsunami-HySEA model for NTHMP tsunami currents benchmarking. Field cases, *Ocean Modelling*, **152**, 101645.

NTHMP, 2012. Proceedings and Results of the 2011 NTHMP Model Benchmarking Workshop, Tech. rep., National Tsunami Hazard Mitigation Program, NOAA Special Report.

Owen, A. & Zhou, Y., 2000. Safe and effective importance sampling, *Journal of the American Statistical Association*, **95**(449), 135–143.

Park, H., Cox, D. T., Lynett, P. J., Wiebe, D. M., & Shin, S., 2013. Tsunami inundation modeling in constructed environments: A physical and numerical comparison of free-surface elevation, velocity, and momentum flux, *Coastal Engineering*, **79**, 9–21.

Tokdar, S. T. & Kass, R. E., 2009. Importance sampling: a review, *WIREs Computational Statistics*, **2**(1), 54–60.

Whiteway, T., 2009. Australian Bathymetry and Topography Grid, June 2009, Tech. rep., Geoscience Australia Record 2009/21.
